# Supplementary material for: Does price regulation affect atorvastatin sales in India? An impact assessment through interrupted time series analysis
Source: BMJ Open. 2019 Jan 24;9(1):e024200. doi: 10.1136/bmjopen-2018-024200 (PMC6347882; doi:10.1136/bmjopen-2018-024200)
Supplement: Supplementary file 1 [file bmjopen-2018-024200supp001.pdf]

Supplementary materials

Indicative calculation of pharmaceutical price cap :

The ceiling prices in DPCO, 2013 were computed using the Market Based Pricing formula[i] as under:

First the Average Price to Retailer, P(s) was computed as under:

P(s) = (Sum of prices to retailer of all the brands and generic versions of the medicine having market share more than or equal to one percent of the total market turnover on the basis of moving annual turnover of that medicine) / (Total number of such brands and generic versions of the medicine having market share more than or equal to one percent of total market turnover on the basis of moving annual turnover for that medicine.)

Then ceiling price was obtained by adding the retailer's margin of 16 percent.

P(c) = P(s).(1+M/100), where
P(s) = Average Price to Retailer for the same strength and dosage of the medicine as calculated in step1 above.
M = % Margin to retailer and its value=16

An indicative calculation of pharmaceutical price cap is presented in Table S1, with an illustration of Atorvastatin market, involving the strength of 5mg and 10mg tablet, underlying the period before and after price fixation. During the period under consideration, there were a total of 33 packs sold in the market involving Atorvastatin 5 mg and 100 plus packs of Atorvastatin 10 mg. Going by principle of only packs with more than or equal to one percent market share in that category, only half and one-fourth of the market gets included in price ceiling calculation for Atorvastatin 5 mg and Atorvastatin 10 mg respectively.

Table S1 : Price to Retailers (PTR) for Atorvastatin

Price Indicators	All packs				Packs with market share>1%			
	2012	2013	2014	2015	2012	2013	2014	2015
Strength: 5mg								
No. of packs	33	33	33	35	18	17	17	16
Median PTR	3.21	3.25	3.09	2.97	3.65	3.71	3.22	3.31
Average PTR	2.94	2.95	2.69	2.75	3.34	3.36	3.02	3.13
Lowest PTR	0.69	0.70	0.79	0.84	1.22	1.33	1.39	1.41
Highest PTR	5.10	4.68	4.96	4.96	5.10	4.68	4.96	4.96
Strength: 10mg								
No. of packs	100	106	109	111	23	25	22	23
Median PTR	4.7	4.9	4.79	4.65	6.41	5.99	4.88	5.11
Average PTR	4.63	4.61	4.28	4.09	5.73	5.15	4.33	4.56

Lowest PTR	0.91	0.95	1.05	0.76	2.21	1.73	1.59	1.65
Highest PTR	8.72	10.47	12.14	10.48	7.98	7.26	5.54	5.67

Several dimensions of price parameters are worth observing from Table S1. In particular, it may be observed that the lowest price-to-retailer (PTR) is markedly lower when all packs are considered, both under 5mg and 10mg. Under price ceiling scenario involving only players with one percent market share, not only the lowest PTR is significantly but median and average PTR involving more than one percent market share is also considerably higher than PTR for all packs.In respect to price ceiling, it is evident that the drug price (measured by price-to-retailer) of Atorvastatin of all packs were significantly lower than packs with one percent market share, whether measured by average or median PTR.

Table S2: Statins in the Indian Pharmaceutical Market

Statins Market	Volumes in billion Sus (%)				Values in INR billions (%)			
	2012	2013	2014	2015	2012	2013	2014	2015
Oveall Statin Market Share	2.50 (0.50)	2.81 (0.56)	3.15 (0.60)	3.56 (0.63)	15.47 (2.16)	16.92 (2.14)	19.41 (2.20)	22.90 (2.25)
FDCs (share in statins market)	0.94 (37.56)	1.14 (40.44)	1.31 (41.76)	1.55 (43.40)	4.56 (29.46)	5.21 (30.79)	6.48 (33.40)	7.92 (34.56)
Plain formulations (share in statins market)	1.56 (62.44)	1.67 (59.56)	1.83 (58.24)	2.02 (56.60)	10.91 (70.54)	11.71 (69.21)	12.93 (66.60)	14.99 (65.44)
Total Market	504357.87	504757.49	523098.93	563619.26	715163.62	788943.16	882423.34	1015741.19

Table S3: Top 5 formulations in the Statins market in terms of values and volumes in the year 2012

	Share in terms of Volumes (%)				Share in terms of Values (%)			
Statins	2012	2013	2014	2015	2012	2013	2014	2015
Atorvastatin Calcium Salt	44.97	40.75	38.10	35.73	48.93	44.96	40.20	38.23
Acetylsalicylic Acid + Atorvastatin Calcium Salt	19.79	20.78	20.86	21.86	5.84	5.55	6.63	6.81
Rosuvastatin Calcium Salt	15.79	17.53	19.15	20.12	19.65	22.66	25.16	26.27
Atorvastatin Calcium Salt + Fenofibrate	6.01	5.45	5.04	4.50	8.55	7.73	7.25	6.37

Fenofibrate + Rosuvastatin Calcium Salt	2.90	3.64	3.97	4.25	4.31	6.13	6.72	7.17
Total share of top 5 statins	89.46	88.14	87.13	86.47	87.28	87.04	85.95	84.84
Other statins	10.54	11.86	12.87	13.53	12.72	12.96	14.05	15.16

Table S4: Absolute policy effect starting the intervention period (model 1 and 2)

	Model 1			Model 2		
Month	Predicted values_actual	Predicted values_counterfactual	Absolute policy effect	Predicted values_actual	Predicted values_counterfactual	Absolute policy effect
Jun-13	28.72	28.84	-0.13			
Jul-13	28.53	28.54	-0.01			
Aug-13	28.34	28.23	0.11	28.47	28.84	-0.37
Sep-13	28.15	27.93	0.23	28.28	28.54	-0.26
Oct-13	27.97	27.62	0.34	28.08	28.23	-0.15
Nov-13	27.78	27.32	0.46	27.89	27.93	-0.04
Dec-13	27.59	27.01	0.58	27.69	27.62	0.07
Jan-14	27.40	26.71	0.7	27.50	27.32	0.18
Feb-14	27.22	26.40	0.82	27.31	27.01	0.29
Mar-14	27.03	26.10	0.93	27.11	26.71	0.40
Apr-14	26.84	25.79	1.05	26.92	26.40	0.52
May-14	26.65	25.49	1.17	26.72	26.10	0.63
Jun-14	26.47	25.18	1.29	26.53	25.79	0.74
Jul-14	26.28	24.88	1.41	26.33	25.49	0.85
Aug-14	26.09	24.57	1.52	26.14	25.18	0.96
Sep-14	25.91	24.26	1.64	25.95	24.88	1.07

Oct-14	25.72	23.96	1.76	25.75	24.57	1.18
Nov-14	25.53	23.65	1.88	25.56	24.26	1.29
Dec-14	25.34	23.35	2	25.36	23.96	1.40
Jan-15	25.16	23.04	2.11	25.17	23.65	1.51
Feb-15	24.97	22.74	2.23	24.97	23.35	1.63
Mar-15	24.78	22.43	2.35	24.78	23.04	1.74
Apr-15	24.59	22.13	2.47	24.59	22.74	1.85
May-15	24.41	21.82	2.59	24.39	22.43	1.96
Jun-15	24.22	21.52	2.7	24.20	22.13	2.07
Jul-15	24.03	21.21	2.82	24.00	21.82	2.18
Aug-15	23.85	20.91	2.94	23.81	21.52	2.29
Sep-15	23.66	20.60	3.06	23.61	21.21	2.40
Oct-15	23.47	20.30	3.18	23.42	20.91	2.51
Nov-15	23.28	19.99	3.29	23.23	20.60	2.63
Dec-15	23.10	19.68	3.41	23.03	20.30	2.74

Tests for Autocorrelation in model 1

Figure 1a.

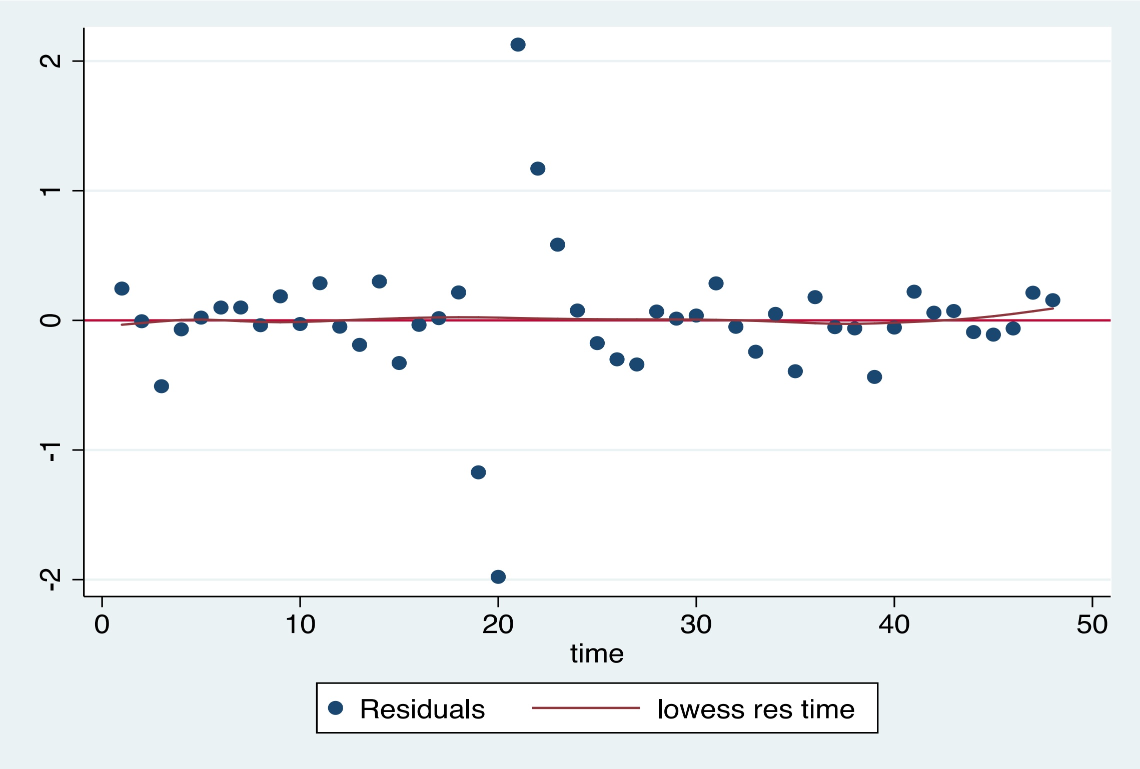

Figure 1b.

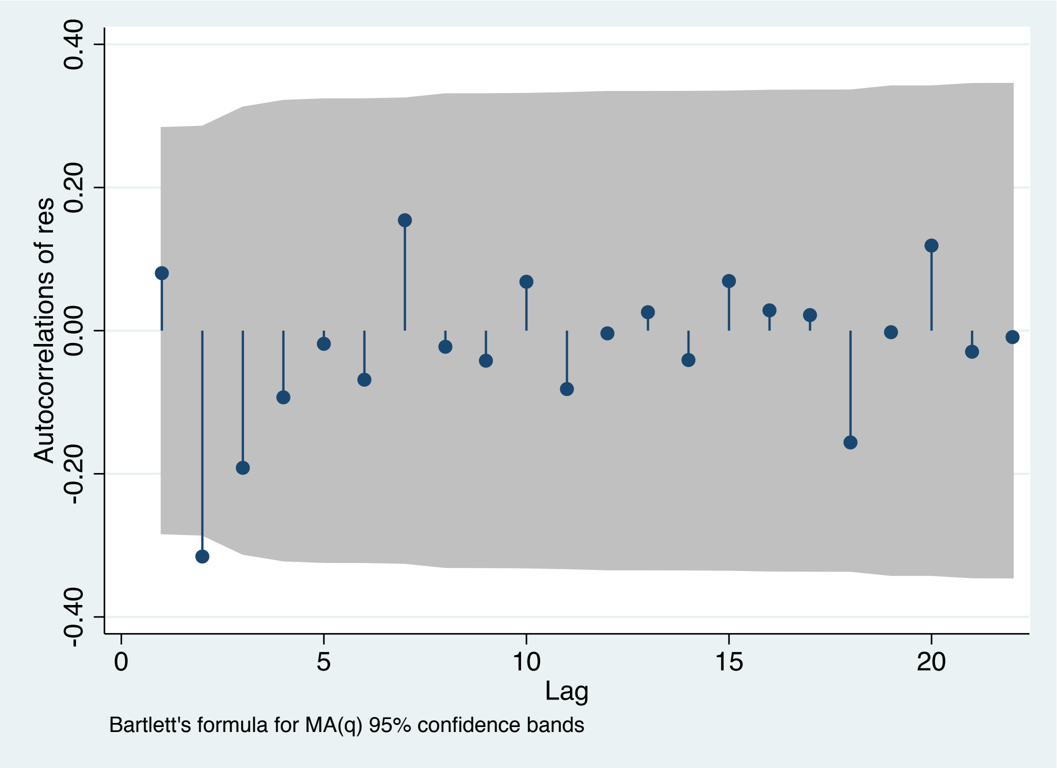

Figure 1c.

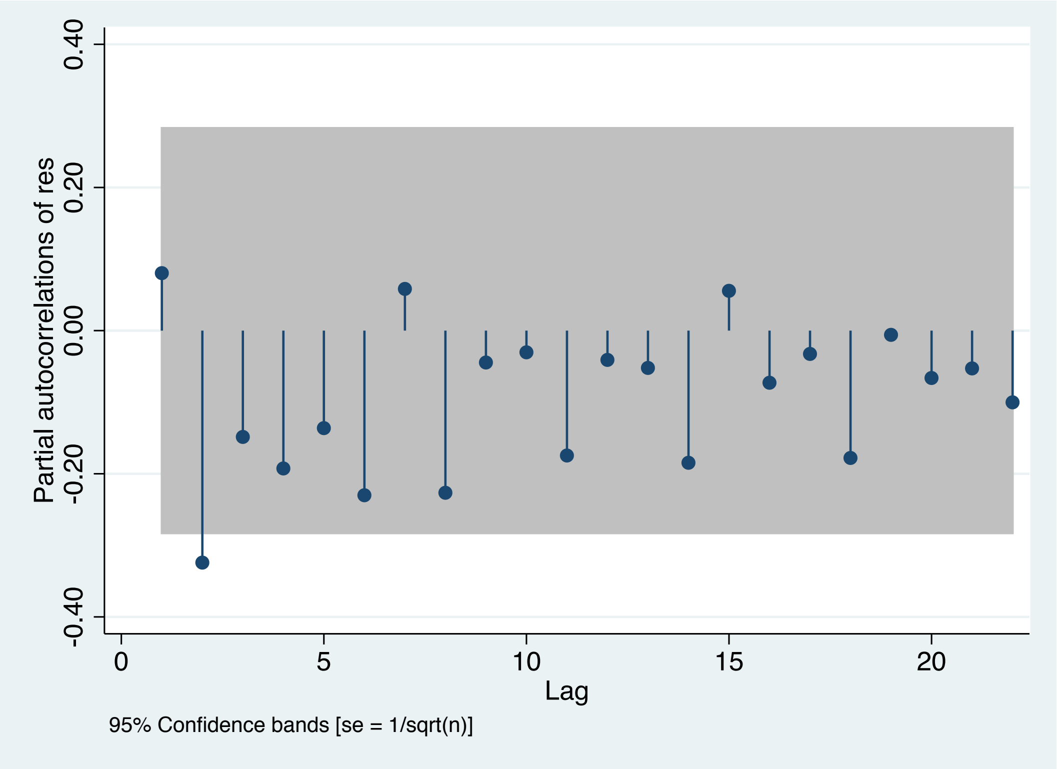

Durbin-Watson d-statistic(4, 48) = 1.833076

Tests for Autocorrelation in model 2

Figure 2a.

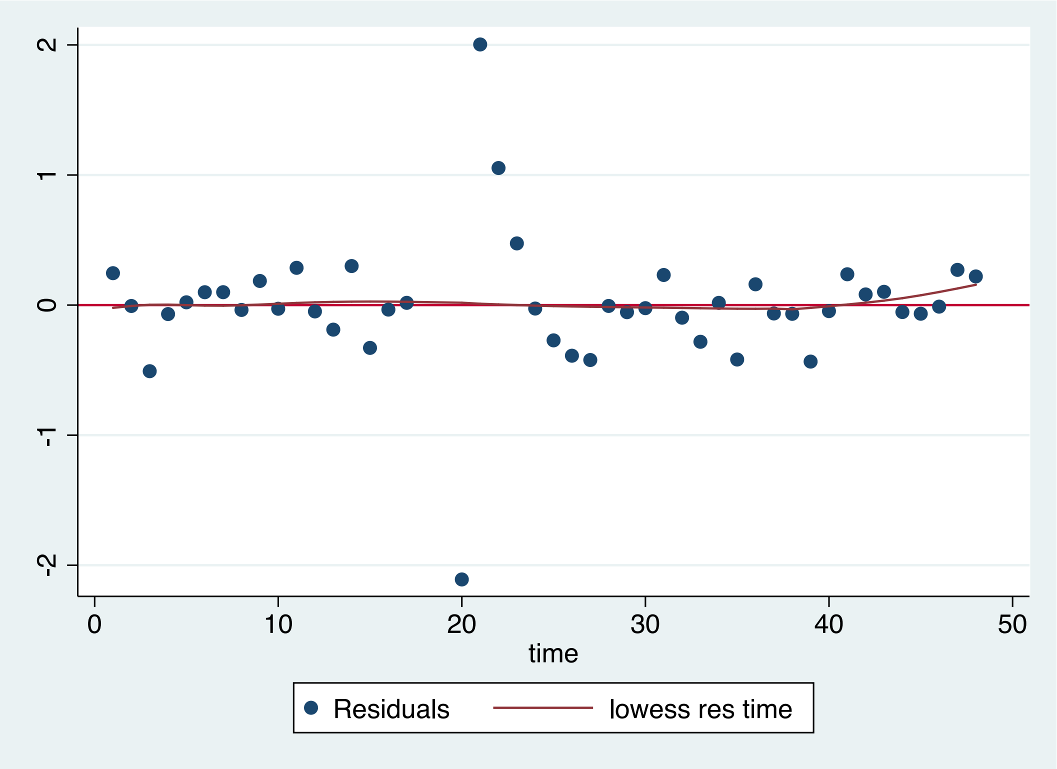

Figure 2b.

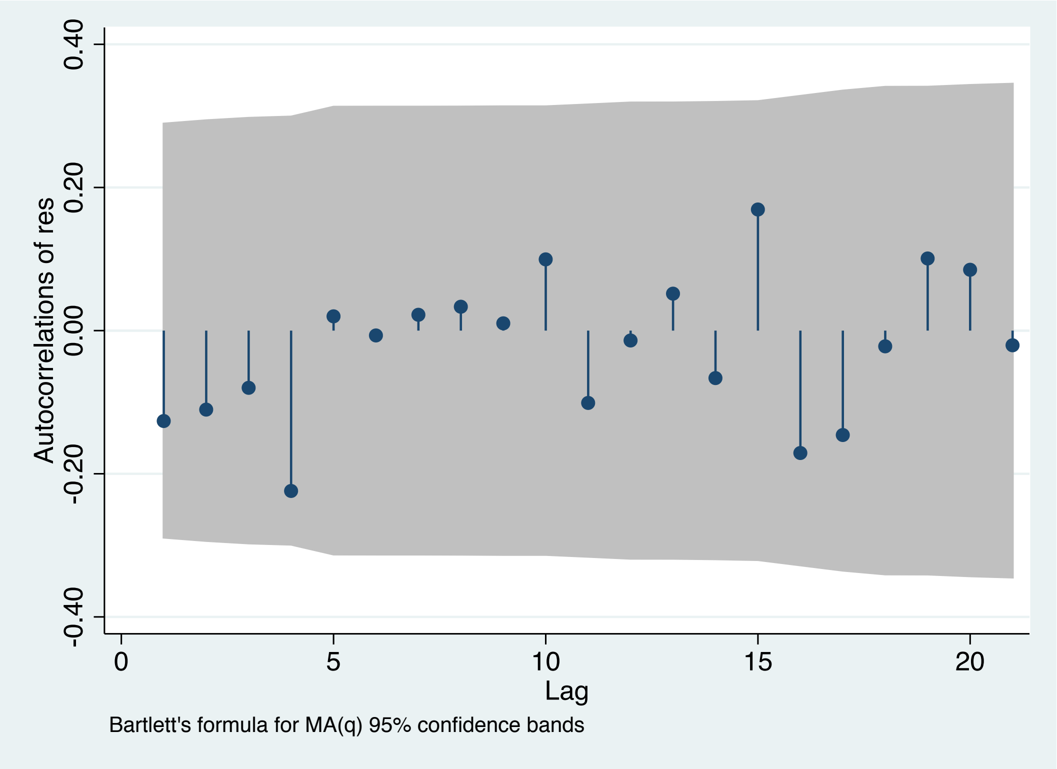

Figure 2c.

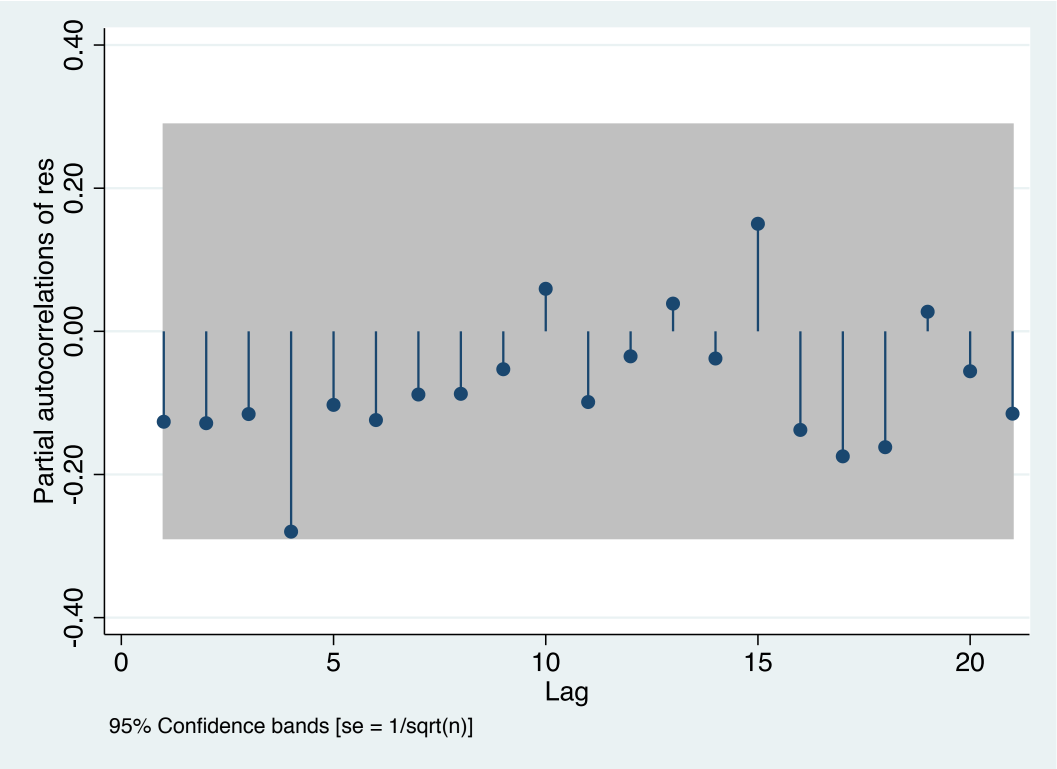

Durbin-Watson d-statistic(4, 46) = 2.243466
