# Supplementary material for: Does price regulation affect atorvastatin sales in India? An impact assessment through interrupted time series analysis
Source: BMJ Open. 2019 Jan 24;9(1):e024200. doi: 10.1136/bmjopen-2018-024200 (PMC6347882; doi:10.1136/bmjopen-2018-024200)
Supplement: Author's manuscript [file bmjopen-2018-024200.draft_revisions.pdf]

BMJ Open

BMJ Open is committed to open peer review. As part of this commitment we make the peer review history of every article we publish publicly available.

When an article is published we post the peer reviewers' comments and the authors' responses online. We also post the versions of the paper that were used during peer review. These are the versions that the peer review comments apply to.

The versions of the paper that follow are the versions that were submitted during the peer review process. They are not the versions of record or the final published versions. They should not be cited or distributed as the published version of this manuscript.

BMJ Open is an open access journal and the full, final, typeset and author-corrected version of record of the manuscript is available on our site with no access controls, subscription charges or pay-per-view fees (http://bmjopen.bmj.com).

If you have any questions on BMJ Open's open peer review process please email info.bmjopen@bmj.com

BMJ Open

Does Price Regulation Affect Atorvastatin Sales in India? An Impact Assessment Through Interrupted Time Series Analysis

Journal:	*BMJ Open*
Manuscript ID	bmjopen-2018-024200
Article Type:	Research
Date Submitted by the Author:	17-May-2018

Complete List of Authors:	Selvaraj, Sakthivel; Public Health Foundation of India<br>Farooqui, Habib; Public Health Foundation of India<br>Mehta, Aashna; Public Health Foundation of India

Keywords:	India, statin, price control, atorvastatin, out of pocket expenditure

SCHOLARONE™
Manuscripts

Title page

Title of the article: Does Price Regulation Affect Atorvastatin Sales in India? An Impact Assessment Through Interrupted Time Series Analysis

Full name, postal address, e-mail and telephone number of the corresponding author:
Dr. Habib Hasan Farooqui, MD
Associate Professor
Address: Public Health Foundation of India, Plot No. 47, Sector 44, Institutional Area, Gurugram, India -122002
Email: drhabibhasan@gmail.com
Phone: +91 9999984673

Full name, department, institution, city and country of all co-authors:
1. Dr. Sakthivel Selvaraj, PhD
Director,
Address: Public Health Foundation of India, Plot No. 47, Sector 44, Institutional Area, Gurugram, India -122002

2. Dr. Habib Hasan Farooqui, MD
Associate Professor,
Address: Public Health Foundation of India, Plot No. 47, Sector 44, Institutional Area, Gurugram, India -122002

3. Ms. Aashna Mehta,
Research Associate,
Address: Public Health Foundation of India, Plot No. 47, Sector 44, Institutional Area, Gurugram, India -122002

For peer review only

For peer review only - http://bmjopen.bmj.com/site/about/guidelines.xhtml

Abstract

Does Price Regulation Affect Atorvastatin Sales in India? An Impact Assessment Through Interrupted Time Series Analysis

Objective: The objective of this study was to examine the impact of medicines price regulation (Drug Price Control Order, 2013) on the sales volume of atorvastatin in the Indian pharmaceutical market.

Setting: All Indian States, January, 2012 to December, 2015

Design: Quasi experimental - Interrupted Time Series Analysis

Data: Pharmaeutical sales audit dataset from IMS Health for the 48 month period from January, 2012 to December, 2015.

Outcome measure : market share of atorvastatin (in percentage) in terms of sales volumes.

Results: We observed that the price regulation notification (DPCO,2013) was associated with an immediate reduction in the average monthly market share of atorvastatin (5 mg and 10 mg) by 0.25 percent (p >0.05; 95% CI: -0.90-0.41) followed by a significant increase in the trend of 0.12 percent (p<0.001; 95% CI: 0.06-0.18) per month. After 31 month of price ceilings notification, the average market share of atorvastatin was 3.41 percent higher than would have been expected had the price ceilings not been notified. In sensitivity analysis, with a control, our findings remain robust, we observed 0.69 percent (p>0.05, 95% CI: -1.54-0.16) immediate post-intervention drop in the average monthly market share of atorvastatin (5 mg and 10 mg) followed by a 0.16 percent (p<0.001; 95% CI: 0.08-0.24) rise in the monthly trend as compared to the change in the control.

Conclusions: Price control as public intervention did improved the sales volume of atorvastatin in India. This policy intervention has potential to improve access to medicines by improving affordability in Indian setting.

Key words: India, price regulation, statin, atorvastatin, out of pocket expenditure

For peer review only - http://bmjopen.bmj.com/site/about/guidelines.xhtml

Article summary

Strengths and limitations of this study

1. To the best of our knowledge this is the first study to report impact of price regulation (DPCO, 2013) on uptake of atorvastatin medicine in India.
2. The use of quasi-experimental research design –interrupted time series analysis with a control is the key strength of this research.
3. Through the use of nationally representative time series data set for 4 year period the study was able to show long term impact of price regulation.
4. We could not analyse the impact of price regulation on utilization of atorvastatin in the public sector due to lack of national level public sector data.

For peer review only

For peer review only - http://bmjopen.bmj.com/site/about/guidelines.xhtml

Does Price Regulation Affect Atorvastatin Sales in India? An Impact Assessment Through Interrupted Time Series Analysis

Background

Medicines, vaccines and other medical supplies remains critical and vital elements of the health system.[1] Apart from being part of one of the vital declaration (Alma Ata Declaration) 1978, access to medicines is also one of the six targets of Millennium Development Goal (MDG) 8 to develop a global partnership for development and continues to be a part of the current Sustainable Development Goals (SDGs), 2030.[2] Despite being the "pharmacy of the global south", a significant share of India's population does not have access to essential medicines.[3] A multi-country study utilizing standard WHO methodology reported that the median availability of a basket of 30 essential medicines in public health facilities ranged from 0-30 percent in six Indian states during 2007.[4] While physical availability of essential medicines in health facilities is a critical indicator, the access framework emphasises the need for examining affordability factor besides availability. This is particularly important in free market economies where availability of medicines may not be an issue but affordability of medicines is, because it translates into poor or no access for people with low purchasing power. In India, although reported availability of cardiovascular medicines was 81% and 89% in rural and urban private pharmacies respectively, yet only 59% of households were able to afford these cardiovascular medicines.[5] Literature suggests that households in India often spend a large share of their out-of-pocket (OOP) expenditure on healthcare.[6] Studies have also reported impoverishment of households because of OOP medicine expenditures.[5 7]

To improve access to healthcare services and reduce financial hardship, national governments resort to various policy instruments, some of them include universal health coverage, publicly funded insurance schemes and pharmaceutical pricing policies.[8] Pharmaceutical pricing policies include policy measures to control medicine prices. These include price control measures like external reference pricing, health technology assessment and cost-plus price setting, measures to control add-on costs in the supply chain (such as controlling wholesaler and dispenser mark-ups) and exemption from taxes and/or tariffs.[9] Literature suggests that most industrialised countries provide medicines free of cost to their citizens or subsidizes them. Government's also uses price control or limiting pharmaceutical profitability as public interventions. Some Organization of Economic Cooperation for Development (OECD) countries for instance, require manufacturer to limit prices in exchange for the subsidies they receive.[10]

India also had progressive drug pricing policies since the year 1979, through the Drug Price Control Orders (DPCO). However, the remit of DPCO had significantly reversed from 1970s when 90% of the market was under price regulation to 2013 by when only

For peer review only - http://bmjopen.bmj.com/site/about/guidelines.xhtml

about 10% of the market was regulated.[11] The Department of Pharmaceuticals, Ministry of Chemicals and Fertilizers on 7th December, 2012 notified National Pharmaceutical Pricing Policy (NPPP), 2012[12] with the objective "to put in place a regulatory framework for pricing of drugs so as to ensure availability of required medicines - 'essential medicines' – at reasonable prices even while providing sufficient opportunity for innovation and competition to support the growth of industry, thereby meeting the goals of employment and shared economic well-being of all." The NPPP 2012 laid down three criteria for price control: (i) regulation of prices based on "essentiality of drugs" (i.e. formulations as listed under the National List of Essential Medicines (NLEM) notified by the Ministry of Health and Family Welfare in 2011)[13], (ii) control of formulation prices only and (iii) market based pricing. The DPCO, 2013 was notified on 15th May, 2013 for the implementation of the NPPP, 2012.

One of the key distinguishing feature of the DPCO, 2013 from earlier avtars of DPCO include implementation of a new mechanism for price control, called Market Based Pricing (MBP).[14] The central idea of a MBP was to compute the ceiling price of a specific formulation under the NLEM by taking a simple average of the prices to retailers (PTRs) of all brands, with a market share equal to or greater than one percent of the overall market for the said formulation and adding a retailer's margin of 16 percent to obtain the maximum retail price (MRP). In earlier DPCOs of 1979, 1986 and 1995, the mechanism of Cost-plus Based Pricing (CBP) was followed for fixing ceiling prices of formulations by taking into account the raw material cost, conversion cost, packaging material cost and the packing charges. A Maximum Allowable Post Manufacturing Expenses (MAPE), were allowed over and above these costs, to the extent of 100 percent.[15] In additon, unlike earlier price control regimes, the new policy sought to apply price capping only on formulations (finished products) rather than the Active Pharmaceutical Ingredient (API). The total number of medicines under price control as outlined in the NLEM, 2011 were 348 which translated into 628 unique strengths and dosage forms of medicines under DPCO, 2013 (for an indicative calculation of pharmaceutical price cap through DPCO, 2013 see supplementary material). Empirical literature suggests that while direct price control policies are effective in reducing prices and are able to control expenditures, they are unable to reduce medicine expenditures in the long run as manufacturers are able to find ways to increase sales of medicine formulations outside price control.[10] In the Indian context, the selective coverage of the policy led to concerns regarding the shift of sales from price controlled medicines to those outside price control but within the same class of medicines as a result of change in marketing priorities of the companies who would have an incentive to market medicines outside price control.

Statins are the medications of choice for cardiovascular risk reduction (i.e. prevention of heart disease, heart attack and stroke).[16] We considered atorvastatin – a class of medicine prescribed to lower blood cholestrol for our analysis because India has the highest burden of cardiovascular disease among developing nations[17] and atorvastatin

For peer review only - http://bmjopen.bmj.com/site/about/guidelines.xhtml

was the only statin under the National List of Essential Medicine, 2011. The price ceilings for two strengths of atorvastatin. (5 mg and 10 mg) were notified on 14th and 21st June, 2013, respectively. The remaining strengths of atorvastatin such as 20mg and 40mg, other statins such as rosuvastatin and simvastatin as well as combinations containing atorvastatin such as atorvastatin + acetylsalicilic acid remain outside price regulation. We hypothesised that on account of new price regulation (DPCO, 2013) there would be a shift in sales of statins from price controlled atorvastatin towards therapeutically equivalent non-price controlled statins. The key objective of this study was to examine the impact of medicines price regulation (Drug Price Control Order, 2013) on the sales volume of atorvastatin in the Indian pharmaceutical market through interuppted time series analysis – a quasi-experimental design. To the best of our knowledge our study is the first attempt from India to generate evidence on the impact of price regulation (DPCO, 2013) on the uptake of medicines used in cardiovascular diseases.

Materials and methods

Data

We used the sales audit dataset from IMS Health (now IQVIA) of the Indian pharmaceutical market for 48 month period from January, 2012 to December, 2015 for this study. IMS Health (now IQVIA) is a for-profit organisation that collects and provides data and information on pharmaceutical market intelligence in over 100 countries around the world . The Indian pharmaceutical sales data is collected from a panel of 5600 stockists across different regions of the country and extrapolated to reflect the sales in the entire country. This comprises the sales made by the stockists to the retailers, hospitals as well as dispensing doctors. The data organizes pharmaceuticals based on anatomical therapeutic classification (ATC) of the European Pharmaceutical Market Research Association (EphMRA). We utilised this information to identify the market for statins in the country.

Outcomes

Our primary outcome measure was market share (in percentage) in terms of sales volumes. Sales volumes are provided in the sales audit dataset in term of standard units (SUs) which are defined by IMS Health (now IQVIA)) as the smallest dose of formulation which can be one tablet or capsule for oral solids, one vial or ampoule for injectables and so on. We computed the monthly share of atorvastatin (5 mg and 10 mg), the statin under the NLEM and hence within the ambit of price regulation, in terms of sales volume in the statins market for the time period under study. Medicines sales was taken as a proxy for consumption of the specific formulation for the purpose of our analysis.

Research design

For peer review only - http://bmjopen.bmj.com/site/about/guidelines.xhtml

We used interrupted time series, a quasi-experimental research design to capture the impact of price ceiling on consumption of atorvastatin (5 mg and 10 mg were notified in under DPCO in June, 2013).[5] We used rosuvastatin (all strengths), a statin outside price regulation as the control to further strengthen our research design.

Statistical analysis

The intervention under study is the notification of price ceilings for atorvastatin (5 mg and 10 mg) in June, 2013. Specifically, the price ceiling was notified for atorvastatin 5 mg on 14th June, 2013 and that for atorvastatin 10 mg was notified on 21st June, 2013. The 48 month period under study was distributed into two segments. The pre-intervention 17 month period from January, 2012 to May, 2013 and the post-intervention period of 31 months starting June, 2013 to December, 2015. We did not take into consideration the period after December 2015 as a new NLEM was notified in this period. Based on the new NLEM, 2015 price celings were subsequently notified for atorvastatin 10 mg, 20 mg and 40 mg in 2016.

We used segmented linear regression analysis[18] to detect the pre-intervention trends, post-intervention level changes, and changes in post-intervention trends relative to the pre-intervention trends in the use of atorvastatin (5mg and 10mg). The dependent variable (Y_t) was 'market share' of atorvastatin (5 mg and 10 mg) in terms of sales volumes while 'time' appeared as an independent variable. We fitted a least square regression line to the two segmnets of the continuous variable time. In addition, we introduced two binary variables to estimate immediate level change after the intervention (variable name: intervention) as well as trend change (variable name: time after intervention) in the market share of regulated atorvastatin (see equation 1 below). The variable 'intervention' was 0 for the pre-intervention period and 1 for the post-intervention period starting June, 2013. Time after intervention was a continuous variable starting June, 2013.[6]

Equation 1:

$$Y_t = \alpha + \beta_1 \text{time}_t + \beta_2 \text{ intervention}_t + \beta_3 \text{ time after intervention}_t + \varepsilon_t$$

The segmented regression helped us statistically estimate the change in the intercept and the slope coefficients between the pre and post-intervention period. We checked the model for autocorrelation with the help of Durbin-Watson statistic, autocorrelation (ac) and partial autocorrelation (pac) estimates and plots of the residuals. We did not detect autocorrelation in our model (see supplementary material for details). Seasonality is unlikely to influence the outcome measure as the group of medicines under study are intended for a chronic condition and meant to be consumed throughout the lifetime of the patients.

For peer review only - http://bmjopen.bmj.com/site/about/guidelines.xhtml

According to the DPCO, 2013, para 13 "… in case of scheduled formulations produced or available in the market before the date of notification of ceiling price, the manufacturers shall ensure within a period of forty-five days of the date of such notification that the maximum retail price of such scheduled formulation does not exceed the ceiling price (plus local taxes as applicable)". The policy was implemented in a phased-in manner as manufacturers got a maximum of 45 days to adjust their maximum retail prices in accordance with the notified ceiling price. Some manufacturers would have been quicker in adjusting their ceiling prices and others would have made use of the full 45-day period. We do not have a way of knowing which manufacturers or how many of them adjusted the ceiling prices in a weeks' time, two weeks' time, three weeks' time and so on. Further, we do not believe that the availability of this information would have impacted our results significantly or that the implementation period was long enough to affect our results. Nonetheless we ran an alternate model (model 2) wherein we excluded the adjustment period of two month from June to August 2013. Inspecting the data also revealed that this period saw a steep decline in the share of price regulated atorvastatin in the statin market which suggests that the suppliers must be withdrawing the stocks from the market to re-label the medicine packs with the revised MRPs.

A counterfactual was introduced into both the model, i.e. trend in consumption of atorvastatin in the post-intervention period had the price ceilings not been notified. It is predicted that in the absence of price ceiling, pre-intervention trend in consumption would have remained constant in the post-intervention period if the price ceilings had not been notified. We computed the difference between the predicted values of the market share of atorvastatin for the actual as well as the counterfactual scenarios to get the estimate of the absolute policy effect. Finally, a control was introduced in the model to strengthen our study design. The marker share of rosuvastatin, a statin completely outside the ambit of price regulation was used as control. Rosuvastatin was chosen as control instead of other statins such as simvastatin, because rosuvastatin is the second highest selling plain formulation in both value and volume terms after atorvastatin. The intention was to control for time varying confounders and other interventions that may have affected the outcome of interest. An interrupted time series design may suffer from both internal and external threats to validity. These threats could be in the form of simultaneously occurring interrventions, seasonal effects, changes in the measurement of the outcome and so on. Therefore, a control group identical to the treatment group followed at the same time as the intervention group, but that did not experience the intervention under study is considered. A comparison of the effect in the intervention group and the control group helps isolate the effect of the intervention under study from other effects occuring at the same time.

We computed the difference in the respective market shares of atorvastatin (5 mg and 10 mg) under price regulation and rosuvastatin, our comparison group. We then ran the above described interrupted time series model with the difference in the market share

For peer review only - http://bmjopen.bmj.com/site/about/guidelines.xhtml

as our new dependent variable. This method had the advantage of simultaneously controlling the pre-intervention differences in the utilization level and trend between the two statins. Once again, two separate models were run – with and without the adjustment period (model 3, see Table 4). We, additionally ran our interrupted time series model on total sales volumes (in SUs) per capita instead of market share for atorvatstatin (5 mg and 10mg) as well statins as a whole to check if the policy has an impact on the sales volumes of the entire statins market.

Results

Our analysis suggest that statin market which was worth INR 22.90 billion in 2015, accounted for 2.25 percent of the Indian pharmaceutical market in terms of sales value and 0.63 percent of the market in terms of sales volume with 3.56 billion standard units (SUs) sold in the same year (Table 1).The market value and volume of statins increased between 2012 and 2015 both in absolute terms as well as in terms of relative share in the overall pharmaceutical market. It is interesting to note that for the time period 2012 to 2015, the share of fixed dose combinations of statins have been rising and that of plain formulation has been falling in terms of both sales values and volumes.

The contribution of atorvastatin in overall statin market, was 44.9 % and 48.9 % in volume and value terms in the year 2012, respectively which went down to 35.7% and 38.2% of the overall statin market by the year 2015 (Table 2). We observed that the market share of regulated atorvastatin (5mg and 10 mg) in the statins market has also been falling in terms of both sales values and volumes for the period 2012 to 2015. For the same period, the market share of another statin, rosuvastatin has been increasing in both value and volume terms. The difference between the market shares of both the statins in terms of sales volume has been narrowing over the study period. In terms of sales values however, the difference between the two market shares turned negative in 2013 and the gap has been widening up to 2015.

Table 1: Statins in the Indian Pharmaceutical Market

**Statins Market**	**Volumes in billion SUs (%)**				**Values in INR billions (%)**			
	**2012**	**2013**	**2014**	**2015**	**2012**	**2013**	**2014**	**2015**
Overall Statins Market (share in the Indian pharmaceutical market)	2.50 (0.50)	2.81 (0.56)	3.15 (0.60)	3.56 (0.63)	15.47 (2.16)	16.92 (2.14)	19.41 (2.20)	22.90 (2.25)

For peer review only - http://bmjopen.bmj.com/site/about/guidelines.xhtml

FDCs (share in statins market)	0.94 (37.56)	1.14 (40.44)	1.31 (41.76)	1.55 (43.40)	4.56 (29.46)	5.21 (30.79)	6.48 (33.40)	7.92 (34.56)
Plain formulations (share in statins market)	1.56 (62.44)	1.67 (59.56)	1.83 (58.24)	2.02 (56.60)	10.91 (70.54)	11.71 (69.21)	12.93 (66.60)	14.99 (65.44)
Total Market	504357.87	504757.49	523098.93	563619.26	715163.62	788943.16	882423.34	1015741.19

Note: SUs refer to Standard Units.

Table 2: Share of atorvastatin 5 mg and 10 mg, other strengths of atorvastatin and rosuvastain in the statins market.

	**Sales volumes (%)**				**Sales values (%)**			
	**2012**	**2013**	**2014**	**2015**	**2012**	**2013**	**2014**	**2015**
**(A)** Atorvastatin (5mg & 10mg) (%)	32.34	28.87	26.29	24.1	25.84	22.4	17.01	15.3
**(B)** Atorvastatin (Other Strengths) (%)	12.63	11.88	11.81	11.64	23.09	22.57	23.19	22.93
**(C=A+B)** Atorvastatin (all strengths)	44.97	40.75	38.1	35.73	48.93	44.96	40.2	38.23
**(D)** Rosuvastatin (all strength) (%)	15.79	17.53	19.15	20.12	19.65	22.66	25.16	26.27
**(E=A-D)** Difference between Atorvastatin (5mg & 10mg) & Rosuvastatin (%)	16.55	11.34	7.14	3.98	6.19	-0.26	-8.15	-10.97

Our results from segmented regression analysis suggest that post intervention, there was an immediate reduction in the average monthly market share of atorvastatin by 0.25 percent but this change in level was insignificant. This was followed by a significant increase in the trend of 0.12 percent ($p<0.001$)) per month. Figure 1a and figure 1b demonstrates fitted values (solid line) of the market share of atorvastatin in the pre and post-intervention period. Additionally, the figure also captures the counterfactual fitted values of market share (dotted line). The counterfactual represents the scenario had the price ceilings for Atorvastatin not been notified during the period under study.

For peer review only - http://bmjopen.bmj.com/site/about/guidelines.xhtml

For peer review only

The vertical line in the figure at June 2013 represents the intervention period which in our case is the month in which price ceilings were notified for Atorvastatin (5 mg and 10 mg) by the NPPA. It is evident from figure 1a and table 3 that the effect was negative for the first couple of months. This was followed by a sustained positive effect for the remaining duration. In December, 2015, the 31st month after the notification of price ceilings, the average market share of Atorvastatin was 3.41 percent higher than would have been expected had the price ceilings not been notified.

We ran another model to check whether the implementation period impacted our results significantly. In model 2 we excluded the period from June 2013 to July 2013. Upon excluding the two-month period, we did not observe a significant modification in our results (see figure 1b). The post-intervention level change continued to be negative and insignificant, although the magnitude increased by 0.11 and the post-intervention trend change slightly reduced by 0.01 but continued to be significant ($p<0.001$). In December 2015, the 31st month after the notification of price ceilings, the average market share of atorvastatin was 2.28 percent higher than would have been expected had the price ceilings not been notified (see table S4 in supplementary material for absolute policy effect from the intervention point June, 2013 up to December, 2015). Excluding the implementation period did not alter our results significantly.

Table 3: Segmented Regression analysis results for drug utilization (atorvastatin 5mg and 10 mg)

	**Model 1**			**Model 2**		
**Variable**	**Coefficients**	**95% conf. interval**		**Coefficients**	**95% conf. interval**	
**Time**	-0.31 (***)	-0.36	-0.25	-0.31 (***)	-0.36	-0.25
**Intervention (level change)**	-0.25	-0.9	0.41	-0.48	-1.12	0.15
**Time after intervention (trend change)**	0.12 (***)	0.06	0.18	0.11 (***)	0.05	0.17
**_Const**	34.34 (***)	33.78	34.9	34.34 (***)	33.8	34.88
**No. of observations**	48 (pre-intervention: 17 and post-intervention: 31)			45 (pre-intervention: 17 and post-intervention: 28)		
**R-squared**	0.9828			0.9763		

Note: *** refers p<0.001 and ** refers to p<0.05

For peer review only - http://bmjopen.bmj.com/site/about/guidelines.xhtml

In order to make our model robust, we introduced a control – another statin - Rosuvastatin (all strengths) which was outside the ambit of price regulation. Table 4 presents result of the segmented regression analysis with the difference in the market shares of atorvastatin (5 mg and 10 mg) and rosuvastatin as the dependent variable. Whereas the relative share of atorvastatin (5 mg and 10 mg) demonstrated a downward trend, the share of rosuvatstatin in the statins market demonstrated an upward trend during the study period (see figure 2). We observed a 0.69 percent ($p>0.05$) immediate post-intervention drop in the average monthly difference in the market share of atorvastatin (5 mg and 10 mg) followed by a 0.16 percent ($p<0.001$) rise in the monthly trend as compared to the change in the control, rosuvastatin. Our results after factoring in the control reiterate robustness of our findings i.e. the post-intervention trend of the average monthly trend of the difference in the market share of atorvastatin (5mg and 10 mg) is positive. Therefore our null hypotheses – that price regulation would lead to a switch away from price regulated atorvastatin (5 mg and 10 mg) towards statins outside price ceiling stands rejected.

In summary, the DPCO 2013 regulation led to a shift towards towards the price regulated atorvastatin (5 mg and 10 mg) from other statins which were not under price regulaion. Finally, we did not observe any effect of the policy on the per capita sales volumes (in SUs) of either atorvastatin (5mg and 10mg) or statins as a whole. The policy did not significantly impact the sales of regulated atorvastatin in absolute terms or statins as a whole. The impact was relative and therefore can be interpreted as a switch towards the regulated atorvastatin.

Table 4: Segmented Regression analysis results for drug utilization (atorvastatin 5mg and 10mg with control)

	**Model 3**		
**Variable**	**Coefficients**	**95% conf. interval**	
**Time**	-0.45 (***)	-0.52	-0.37
**Intervention (level change)**	-0.69	-1.54	0.16
**Time after intervention (trend change)**	0.16 (***)	0.08	0.24
**_Const**	19.49 (***)	18.76	20.22
**No. of observations**	48 (pre-intervention: 17 and post-intervention: 31)		
**R-squared**	0.9808		

Note: *** refers p<0.001; ** refers to p<0.05

Discussion

We have generated robust evidence on the impact of price regulation policy (DPCO, 2013) of Government of India on sales of one of the essential medicine under NLEM - atorvastatin (5mg and 10 mg) through use of interrupted time series analysis –a quasi

For peer review only - http://bmjopen.bmj.com/site/about/guidelines.xhtml

experimental design. The use of interrupted time series analysis to measue the policy impact by comparing pre-interventon verus post-intervention trend has increased in recent past especially to assess impact of health related interventions.[19-25] Our research design, analytic approach and reporting conform to well establish methodological standards.[26 27]

We observed that the baseline trend for average monthly atorvastattin sales was a declining trend during the study period (January 2012 to Decemebr 2015). Some of the reasons for such trend includes prescription preferences, availability of therapeutically substitutable products and marketing practices of manufactures. However, the sales of the price controlled formulation of atorvastatin (5mg and 10mg) relative to other statins increased after the ceiling price notification in June 2013. Further, after a initial downward shift in sales, which could have resulted from the recall of existing packs of atorvastatin from retail market for repackaging and reprinting the new maximum retail prices (MRPs)[28] as mandated by NPPA in NPPP, 2012 and DPCO 2013, there was sustained posititive effect of regulation as reflected in increasing monthly sales. The sustained positive effect may have resulted primarily because of price reductions leading to increased uptake of the price regulated atorvastatin (5mg and 10mg) on account of increased affordability.

Empirical literature suggests that generally, direct price control policies are usually unable to reduce medicine expenditures as manufacturers are able to find ways to increase sales of medicine formulations outside regulation.[10] Recent research on price control of anti-hypertensive medicines from Korea reported some unintended effect of the policy i.e. drug price reduction resulted in drug overutilisation and use of prohibited combinations. Also, the utilisation of drugs, which was not affected by the drug price reduction, increased by 12.3%.[29] In addtion, literature suggest various kinds of demand side and supply side price control policy interventions, each with its own benefits and limitations. WHO guidelines on pharmaceutical pricing policies also suggest that at any given point of time national governments should employ a judicious mix of different policy instruments to control medicine prices and expenditure.[9]

Our study has some limitations. We have evaluated the impact of price regulation on only one market, our findings are not representative for other medicines or formulation under price control. However, it should be noted that the impact of the policy could vary across segments as the market dynamics in terms of the number of players, relative competition, the behaviour of the players in different segments varies, as does consumer behaviour, therefore there is merit in studying individual markets separately. An important limitation of our study is that the IMS sales audit data was representative only for private sector medicines sales for outpatient care, hence we could not comment on impact of price regulation on atorvastatin utilization in public sector. Further, the medicines sales has been used as proxy for utilization. We have also not evaluated the effect of increased utilization of atorvastatin on health outcomes.

For peer review only - http://bmjopen.bmj.com/site/about/guidelines.xhtml

Conclusions

The effectiveness of pharmaceutical pricing policies depends on the scope and mechanisms around which the regulation is designed. It has been argued that partial price regulation involving one particular drug formulation without considering other equivalent therapeutic formulation may result in switch away from price regulated products to unregulated pharmaceutical products.[30] On the contrary, the evidence emerging from our study using segmented regression analysis model controlling for confounders, indicates that the relative consumption of price controlled atorvastation (5mg and 10mg) has increased in the post-intervention period. The NPPA has notified the Drugs (Price Control) Amendment Order, 2016 [31] after the announcement of National List of Essential Medicines (NLEM), 2015. Now, few more strengths of atorvastatin (20mg and 40mg) are under price control. To ascertain the effect of increased coverage of price regulation, another segmented regression analysis should be conducted after sufficient time period has lapsed after implementation of Drugs (Price Control) Amendment Order, 2016. More research is needed to measure the impact of price regulation in other markets on the basis of on other medicines which are part of NLEM 2015.

Contributions: SS, HHF, and AM conceived the idea, designed the analysis, conducted data analysis and wrote the first draft of the paper. SS, HHF, and AM conducted the literature review and the interpretation of the results. SS, HHF, and AM revised and edited the manuscript to its final stages. All the authors approved the final manuscript version.

Acknowledgments: The authors would like to thank Dr. Anup Karan (Asscoiate Professor, Indian Institute of Public Health, Public Health Foundation of India) for his critical and constructive comments on the methodology.

Competing Interests: We declare no conflict of interest.

Funding: Habib Hasan Farooqui is supported by a Wellcome Trust Capacity Strengthening Strategic Award (084754/Z/08/A) to the Public Health Foundation of India and a consortium of UK universities. The funders had no role in study design, data collection and analysis, decision to publish, or preparation of the manuscript.

Data sharing: The IMS health data is available on request, at the approval of IMS Health.

Ethical approval: Ethical approval for this study was not needed. The study used only anonymised data from secondary sources.

Reference:

For peer review only - http://bmjopen.bmj.com/site/about/guidelines.xhtml

1. Bigdeli M, Jacobs B, Tomson G, et al. Access to medicines from a health system perspective. *Health policy and planning* 2013;28(7):692-704. doi: 10.1093/heapol/czs108
2. Gotham D, Onarheim KH, Barber MJ. How the MDGs gave up on measuring access to medicines. *The Lancet Global health* 2016;4(5):e296-7. doi: 10.1016/S2214-109X(16)00066-8
3. WHO. THE WORLD MEDICINES SITUATION. Geneva: World Health Organization, 2004.
4. Cameron A, Ewen M, Ross-Degnan D, et al. Medicine prices, availability, and affordability in 36 developing and middle-income countries: a secondary analysis. *Lancet* 2009;373(9659):240-9. doi: 10.1016/S0140-6736(08)61762-6
5. Khatib R, McKee M, Shannon H, et al. Availability and affordability of cardiovascular disease medicines and their effect on use in high-income, middle-income, and low-income countries: an analysis of the PURE study data. *Lancet* 2016;387(10013):61-9. doi: 10.1016/S0140-6736(15)00469-9
6. Karan A, Selvaraj S, Mahal A. Moving to universal coverage? Trends in the burden of out-of-pocket payments for health care across social groups in India, 1999-2000 to 2011-12. *PloS one* 2014;9(8):e105162. doi: 10.1371/journal.pone.0105162
7. Rao KD, Bhatnagar A, Murphy A. Socio-economic inequalities in the financing of cardiovascular & diabetes inpatient treatment in India. *Indian J Med Res* 2011;133:57-63.
8. Veronika J Wirtz HVH, Andrew L Gray, Maryam Bigdeli, Cornelis P de Joncheere, Margaret A Ewen, et. al. Essential medicines for universal health coverage. *Lancet* 2016;389:403-76.
9. WHO. WHO Guideline on Country Pharmaceutical Pricing Policies. Geneva: World Health Organization, 2013.
10. MSH. Pharmaceutical Pricing Policy: Management Sciences for Health, 2012.
11. Sakthivel Selvaraj HHF. Legitimising Unaffordable Medicine Prices? Draft Drug Price Policy 2011. *Economic and Political Weekly* 2012;Vol. 47,(Issue No. 46,)
12. National Pharmaceutical Pricing Policy 2012. India: Government of India, 2012.
13. MOHFW. National List of Essential Medicines (NLEM) 2011. New Delhi: Ministry of Health and Family Welfare, 2011.
14. India Go. Drug Price Control Order 2013. In: Department of Pharmaceuticals MoCaF, ed. India, 2013.
15. India Go. Drug Price Control Order 1995. In: Department of Pharmaceuticals MoCaF, ed. India, 1995.
16. Niteesh K. Choudhry SD, William H. Shrank, Jennifer M. Polinski, Christina E. Stark, Rajeev Gupta, Dorairaj Prabhakaran, Gregory Brill and Prabhat Jha. Despite Increased Use And Sales Of Statins In India, Per Capita Prescription Rates Remain Far Below High-Income Countries. *Health Affairs,* 2014;33(2):273-82.
17. Patel V CS, Chisholm D, Ebrahim S, Gopalakrishna G, Mathers C, et al. . Chronic diseases and injuries in India. . *Lancet* 2011;377(9763):413-28.

For peer review only - http://bmjopen.bmj.com/site/about/guidelines.xhtml

18. Singal GL, Nanda A, Kotwani A. A comparative evaluation of price and quality of some branded versus branded-generic medicines of the same manufacturer in India. *Indian journal of pharmacology* 2011;43(2):131-6. doi: 10.4103/0253-7613.77344
19. Yoo K-B LS, Park S, et al. . Effects of drug price reduction and prescribing restrictions on expenditures and utilisation of antihypertensive drugs in Korea. . *BMJ Open* 2015;5
20. Garabedian LF R-DD, Ratanawijitrasin S, et al. Impact of universal health insurance coverage in Thailand on sales and market share of medicines for non-communicable diseases:ban interrupted time series study. . *BMJ Open* 2012;2
21. Leopold C M-TA, Seyfang L, Vogler S, Joncheere KD, Laing OR, Leufkens H. . Impact of External Price Referencing on Medicine Prices – a Price Comparison among 14 European countries. . *Southern Med Review* 2012;5(2):34-41.
22. Gianfranco Damiani BF, Angela Anselmi, Caterina Bianca Neve Aurora Bianchi, Giulia Silvestrini, et. al. The impact of Regional co-payment and National reimbursement criteria on statins use in Italy: an interrupted time-series analysis. *BMC health services research* 2014;14(6)
23. Evangelos Kontopantelis TD, David A Springate,Iain Buchan,David Reeves. Regression based quasi-experimental approach when randomisation is not an option: interrupted time series analysis. *Bmj* 2015;350
24. Lagarde M. How to do (or not to do) . . . Assessing the impact of a policy change with routine longitudinal data. *Health policy and planning* 2011
25. James Lopez Bernal SC, Antonio Gasparrini. Interrupted time series regression for the evaluation of public health interventions: a tutorial. *International Journal of Epidemiology,* 2016
26. Wagner AK, Soumerai SB, Zhang F, et al. Segmented regression analysis of interrupted time series studies in medication use research. *J Clin Pharm Ther* 2002;27(4):299-309.
27. Racquel Jandoca AMB, Muhammad Mamdania,b,c, Linda E. L evesqued,, Cadarettea SM. Interrupted time series analysis in drug utilization research is increasing: systematic review and recommendations. *Journal of Clinical Epidemiology* 2015;68:950-56.
28. NPPA. Compendium of notified ceiling prices of schedules drug - 2015: National Pharmaceutical Pricing Authority, 2015.
29. Kwon HY, Hong JM, Godman B, et al. Price cuts and drug spending in South Korea: the case of antihyperlipidemic agents. *Health policy* 2013;112(3):217-26. doi: 10.1016/j.healthpol.2013.08.011
30. Ajay Bhaskarabhatla CC, Priyatam Anurag and Enrico Pennings. Mitigating regulatory impact: the case of partial price controls on metformin in India. *Health policy and planning* 2016
31. Bureau PI. Ceiling prices of 464 formulation fixed after announcement of NLEM, 2015 and Revised Schedule-I, resulting in savings of Rs 2288 crore for consumers 2016 [Available from: http://pib.nic.in/newsite/PrintRelease.aspx?relid=151146 accessed June 2017.

For peer review only - http://bmjopen.bmj.com/site/about/guidelines.xhtml

Figure legends:

Figure 1a: Fitted values of the market share of atorvastatin 5 mg and 10 mg - actual and counterfactual (Model 1)
Note: ms refers to the market share of atorvastatin (5mg and 10mg)

Figure 1b: Fitted values of the market share of atorvastatin 5 mg and 10 mg - actual and counterfactual (Model 2)
Note: ms_ator refers to the market share of atorvastatin (5mg and 10mg)

Figure 2: Fitted values of the difference in the market share of atorvastatin (5 mg, 10 mg) and Rosuvastatin (all strengths) - actual and counterfactual
Note: ms_diff refers to the difference between the market share of atorvastatin (5mg and 10mg) and rosuvatstain (all strength); ms_ator refers to the market share of atorvastatin (5mg and 10mg); and ms_rosuv refers to the market share of rosuvatstain (all strength)

For peer review only

For peer review only - http://bmjopen.bmj.com/site/about/guidelines.xhtml

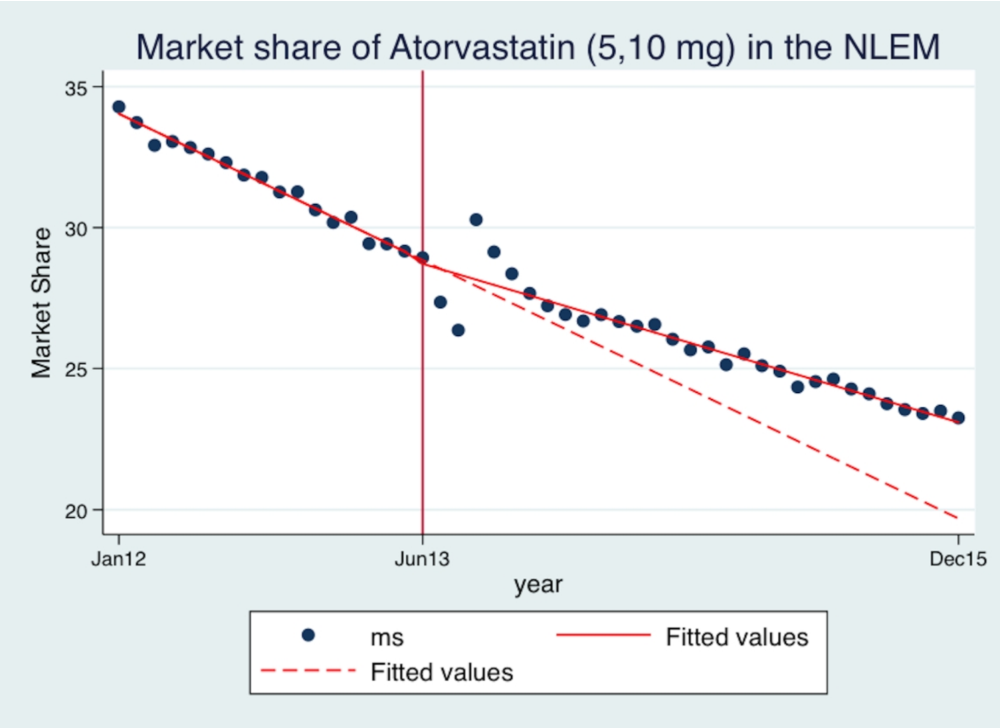

Figure 1a: Fitted values of the market share of atorvastatin 5 mg and 10 mg - actual and counterfactual (Model 1)
Note: ms refers to the market share of atorvastatin (5mg and 10mg)

139x101mm (300 x 300 DPI)

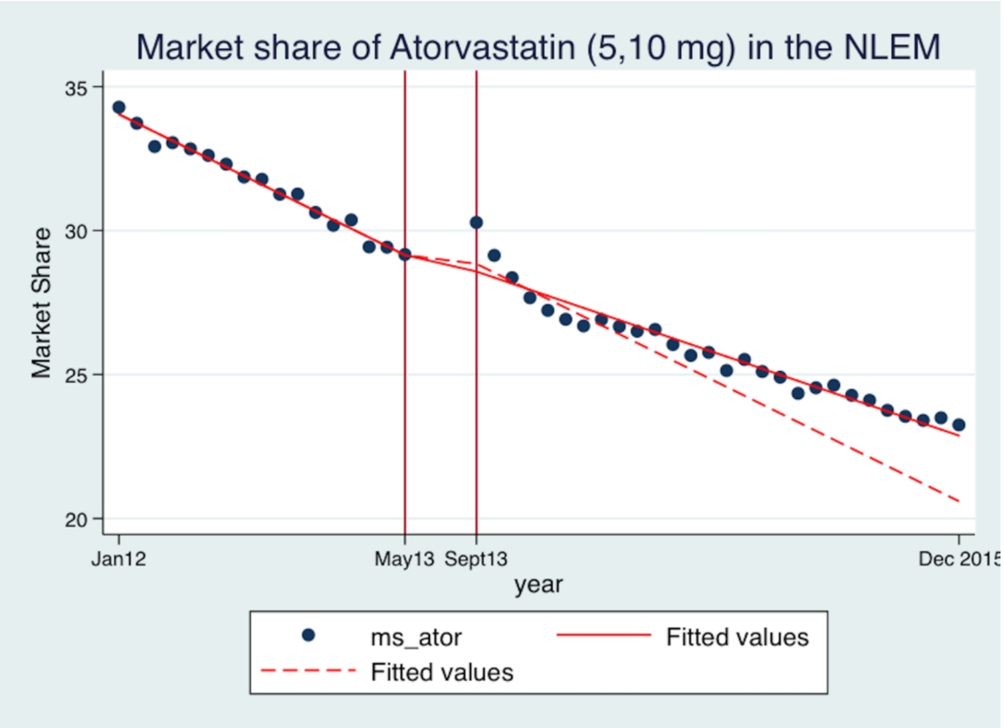

Figure 1b: Fitted values of the market share of atorvastatin 5 mg and 10 mg - actual and counterfactual (Model 2)
Note: ms_ator refers to the market share of atorvastatin (5mg and 10mg)

139x101mm (300 x 300 DPI)

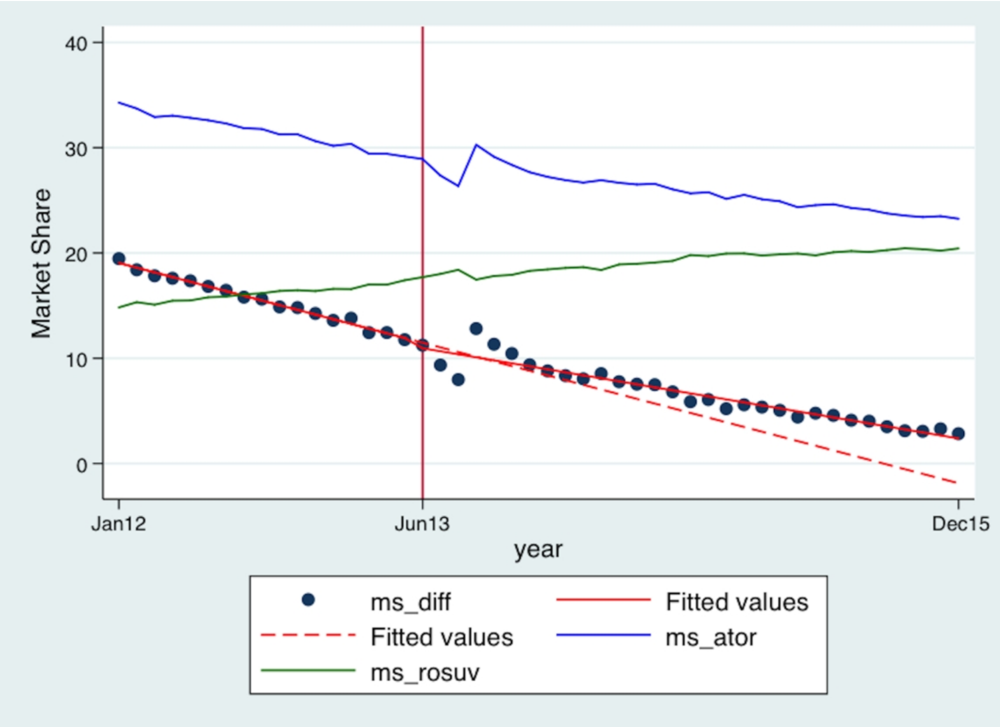

Figure 2: Fitted values of the difference in the market share of atorvastatin (5 mg, 10 mg) and Rosuvastatin (all strengths) - actual and counterfactual
Note: ms_diff refers to the difference between the market share of atorvastatin (5mg and 10mg) and rosuvatstain (all strength); ms_ator refers to the market share of atorvastatin (5mg and 10mg); and ms_rosuv refers to the market share of rosuvatstain (all strength)

139x101mm (300 x 300 DPI)

Supplementary materials

Indicative calculation of pharmaceutical price cap :

The ceiling prices in DPCO, 2013 were computed using the Market Based Pricing formula[i] as under:

First the Average Price to Retailer, P(s) was computed as under:

P(s) = (Sum of prices to retailer of all the brands and generic versions of the medicine having market share more than or equal to one percent of the total market turnover on the basis of moving annual turnover of that medicine) / (Total number of such brands and generic versions of the medicine having market share more than or equal to one percent of total market turnover on the basis of moving annual turnover for that medicine.)

Then ceiling price was obtained by adding the retailer's margin of 16 percent.

P(c) = P(s).(1+M/100), where
P(s) = Average Price to Retailer for the same strength and dosage of the medicine as calculated in step1 above.
M = % Margin to retailer and its value=16

An indicative calculation of pharmaceutical price cap is presented in Table S1, with an illustration of Atorvastatin market, involving the strength of 5mg and 10mg tablet, underlying the period before and after price fixation. During the period under consideration, there were a total of 33 packs sold in the market involving Atorvastatin 5 mg and 100 plus packs of Atorvastatin 10 mg. Going by principle of only packs with more than or equal to one percent market share in that category, only half and one-fourth of the market gets included in price ceiling calculation for Atorvastatin 5 mg and Atorvastatin 10 mg respectively.

Table S1 : Price to Retailers (PTR) for Atorvastatin

Price Indicators	All packs				Packs with market share>1%			
	2012	2013	2014	2015	2012	2013	2014	2015
Strength: 5mg								
No. of packs	33	33	33	35	18	17	17	16
Median PTR	3.21	3.25	3.09	2.97	3.65	3.71	3.22	3.31
Average PTR	2.94	2.95	2.69	2.75	3.34	3.36	3.02	3.13
Lowest PTR	0.69	0.70	0.79	0.84	1.22	1.33	1.39	1.41
Highest PTR	5.10	4.68	4.96	4.96	5.10	4.68	4.96	4.96
Strength: 10mg								
No. of packs	100	106	109	111	23	25	22	23
Median PTR	4.7	4.9	4.79	4.65	6.41	5.99	4.88	5.11
Average PTR	4.63	4.61	4.28	4.09	5.73	5.15	4.33	4.56

For peer review only

Lowest PTR	0.91	0.95	1.05	0.76	2.21	1.73	1.59	1.65
Highest PTR	8.72	10.47	12.14	10.48	7.98	7.26	5.54	5.67

Several dimensions of price parameters are worth observing from Table S1. In particular, it may be observed that the lowest price-to-retailer (PTR) is markedly lower when all packs are considered, both under 5mg and 10mg. Under price ceiling scenario involving only players with one percent market share, not only the lowest PTR is significantly but median and average PTR involving more than one percent market share is also considerably higher than PTR for all packs.In respect to price ceiling, it is evident that the drug price (measured by price-to-retailer) of Atorvastatin of all packs were significantly lower than packs with one percent market share, whether measured by average or median PTR.

Table S2: Statins in the Indian Pharmaceutical Market

Statins Market	Volumes in billion Sus (%)				Values in INR billions (%)			
	2012	2013	2014	2015	2012	2013	2014	2015
Oveall Statin Market Share	2.50 (0.50)	2.81 (0.56)	3.15 (0.60)	3.56 (0.63)	15.47 (2.16)	16.92 (2.14)	19.41 (2.20)	22.90 (2.25)
FDCs (share in statins market)	0.94 (37.56)	1.14 (40.44)	1.31 (41.76)	1.55 (43.40)	4.56 (29.46)	5.21 (30.79)	6.48 (33.40)	7.92 (34.56)
Plain formulati ons (share in statins market)	1.56 (62.44)	1.67 (59.56)	1.83 (58.24)	2.02 (56.60)	10.91 (70.54)	11.71 (69.21)	12.93 (66.60)	14.99 (65.44)
Total Market	504357.87	504757.49	523098.93	563619.26	715163.62	788943.16	882423.34	1015741.19

Table S3: Top 5 formulations in the Statins market in terms of values and volumes in the year 2012

	Share in terms of Volumes (%)				Share in terms of Values (%)			
Statins	2012	2013	2014	2015	2012	2013	2014	2015
Atorvastatin Calcium Salt	44.97	40.75	38.10	35.73	48.93	44.96	40.20	38.23
Acetylsalicylic Acid + Atorvastatin Calcium Salt	19.79	20.78	20.86	21.86	5.84	5.55	6.63	6.81
Rosuvastatin Calcium Salt	15.79	17.53	19.15	20.12	19.65	22.66	25.16	26.27
Atorvastatin Calcium Salt + Fenofibrate	6.01	5.45	5.04	4.50	8.55	7.73	7.25	6.37

For peer review only

Fenofibrate + Rosuvastatin Calcium Salt	2.90	3.64	3.97	4.25	4.31	6.13	6.72	7.17
Total share of top 5 statins	89.46	88.14	87.13	86.47	87.28	87.04	85.95	84.84
Other statins	10.54	11.86	12.87	13.53	12.72	12.96	14.05	15.16

Table S4: Absolute policy effect starting the intervention period (model 1 and 2)

	Model 1			Model 2		
Month	Predicted values_actual	Predicted values_counterfactual	Absolute policy effect	Predicted values_actual	Predicted values_counterfactual	Absolute policy effect
Jun-13	28.72	28.84	-0.13			
Jul-13	28.53	28.54	-0.01			
Aug-13	28.34	28.23	0.11	28.47	28.84	-0.37
Sep-13	28.15	27.93	0.23	28.28	28.54	-0.26
Oct-13	27.97	27.62	0.34	28.08	28.23	-0.15
Nov-13	27.78	27.32	0.46	27.89	27.93	-0.04
Dec-13	27.59	27.01	0.58	27.69	27.62	0.07
Jan-14	27.40	26.71	0.7	27.50	27.32	0.18
Feb-14	27.22	26.40	0.82	27.31	27.01	0.29
Mar-14	27.03	26.10	0.93	27.11	26.71	0.40
Apr-14	26.84	25.79	1.05	26.92	26.40	0.52
May-14	26.65	25.49	1.17	26.72	26.10	0.63
Jun-14	26.47	25.18	1.29	26.53	25.79	0.74
Jul-14	26.28	24.88	1.41	26.33	25.49	0.85
Aug-14	26.09	24.57	1.52	26.14	25.18	0.96
Sep-14	25.91	24.26	1.64	25.95	24.88	1.07

For peer review only

Oct-14	25.72	23.96	1.76	25.75	24.57	1.18
Nov-14	25.53	23.65	1.88	25.56	24.26	1.29
Dec-14	25.34	23.35	2	25.36	23.96	1.40
Jan-15	25.16	23.04	2.11	25.17	23.65	1.51
Feb-15	24.97	22.74	2.23	24.97	23.35	1.63
Mar-15	24.78	22.43	2.35	24.78	23.04	1.74
Apr-15	24.59	22.13	2.47	24.59	22.74	1.85
May-15	24.41	21.82	2.59	24.39	22.43	1.96
Jun-15	24.22	21.52	2.7	24.20	22.13	2.07
Jul-15	24.03	21.21	2.82	24.00	21.82	2.18
Aug-15	23.85	20.91	2.94	23.81	21.52	2.29
Sep-15	23.66	20.60	3.06	23.61	21.21	2.40
Oct-15	23.47	20.30	3.18	23.42	20.91	2.51
Nov-15	23.28	19.99	3.29	23.23	20.60	2.63
Dec-15	23.10	19.68	3.41	23.03	20.30	2.74

For peer review only

Tests for Autocorrelation in model 1

Figure 1a.

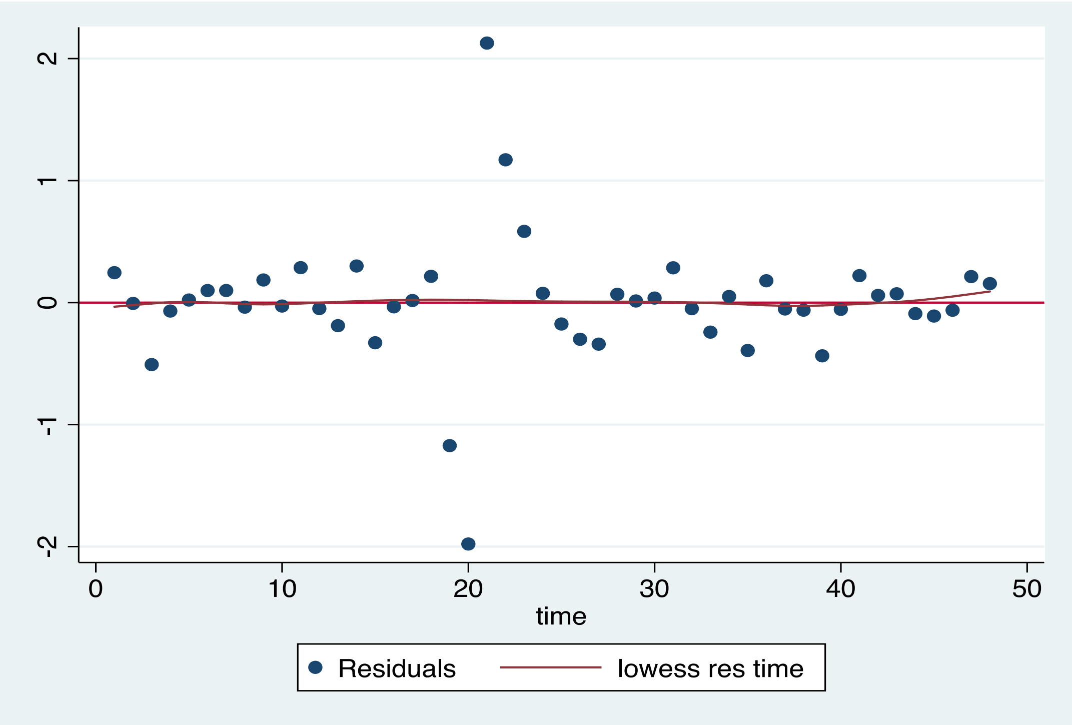

Figure 1b.

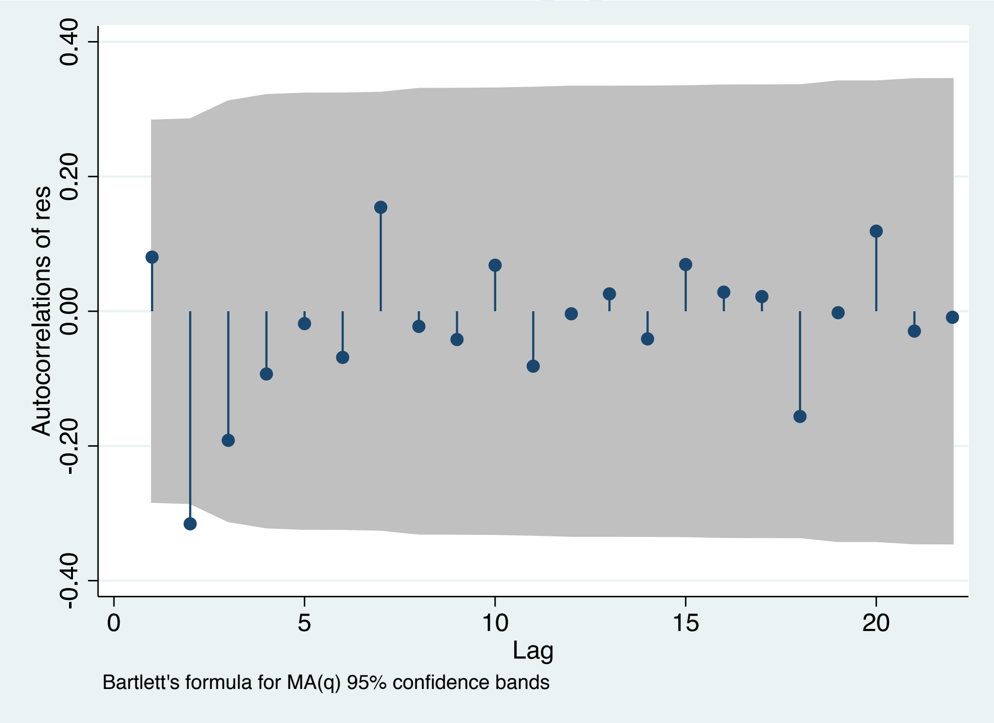

Figure 1c.

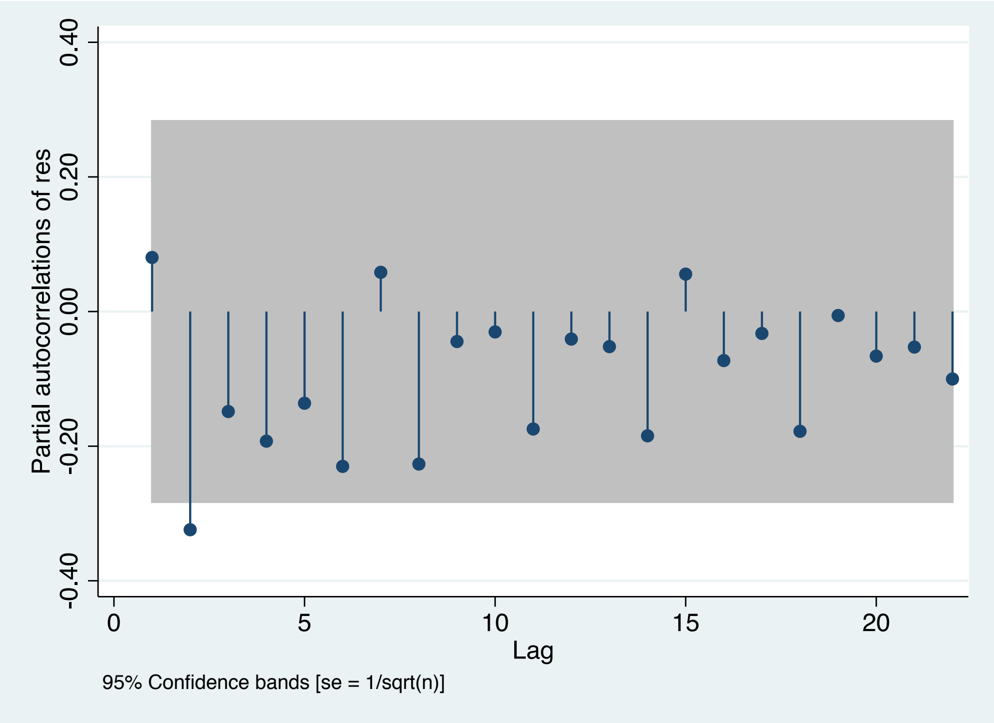

Durbin-Watson d-statistic(4, 48) = 1.833076

Tests for Autocorrelation in model 2

Figure 2a.

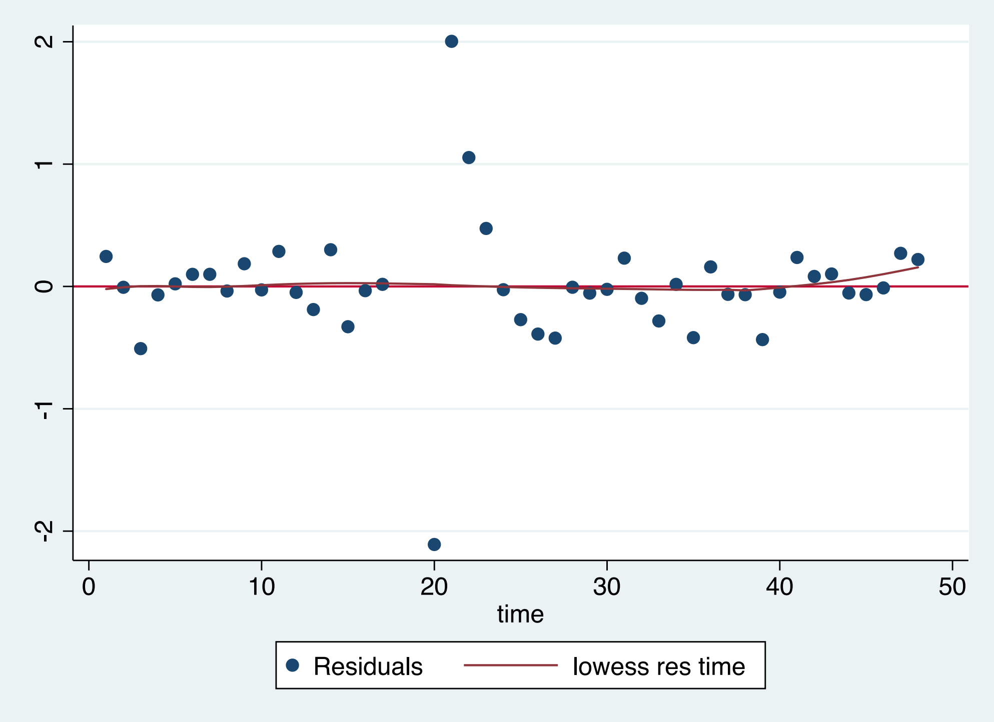

Figure 2b.

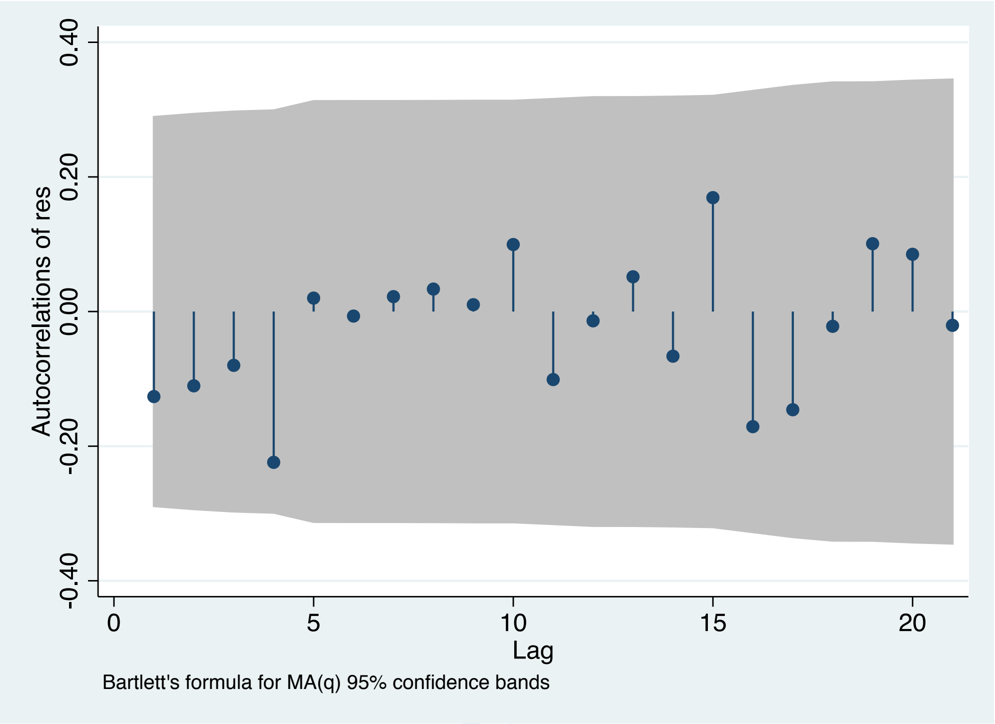

Figure 2c.

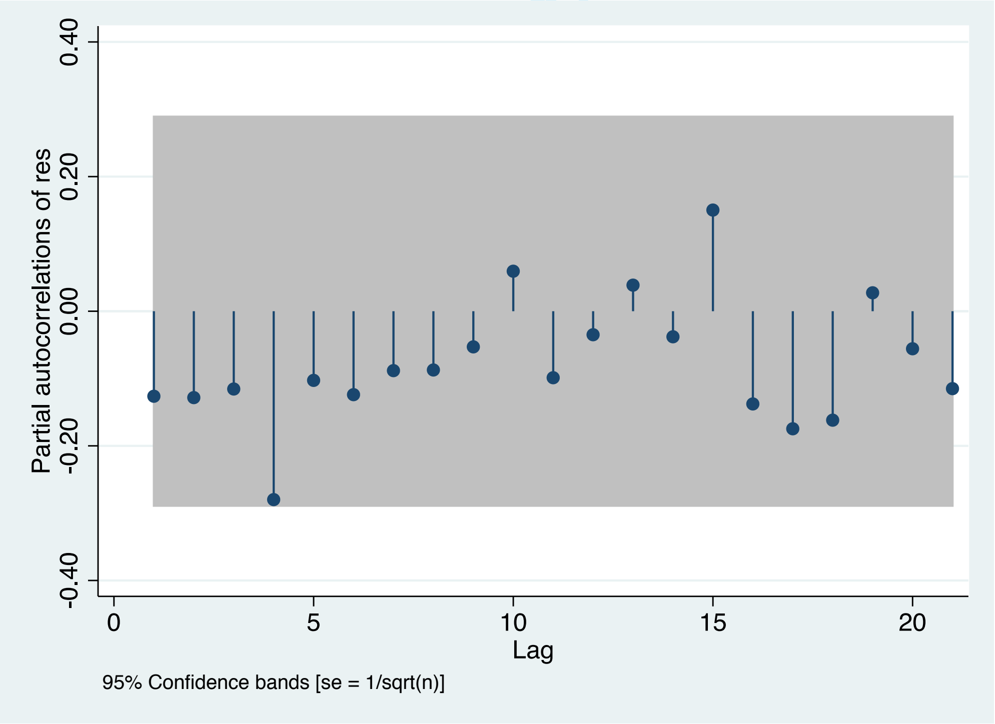

Durbin-Watson d-statistic(4, 46) = 2.243466

Does Price Regulation Affect Atorvastatin Sales in India? An Impact Assessment Through Interrupted Time Series Analysis

Journal:	*BMJ Open*
Manuscript ID	bmjopen-2018-024200.R1
Article Type:	Research
Date Submitted by the Author:	08-Sep-2018
Complete List of Authors:	Selvaraj, Sakthivel; Public Health Foundation of India<br>Farooqui, Habib; Public Health Foundation of India<br>Mehta, Aashna; Public Health Foundation of India
**Primary Subject Heading**:	Health economics
Secondary Subject Heading:	Health economics
Keywords:	India, statin, price control, atorvastatin, out of pocket expenditure

Title page

Title of the article: Does Price Regulation Affect Atorvastatin Sales in India? An Impact Assessment Through Interrupted Time Series Analysis

Full name, postal address, e-mail and telephone number of the corresponding author:
Dr. Habib Hasan Farooqui, MD
Associate Professor
Address: Public Health Foundation of India, Plot No. 47, Sector 44, Institutional Area, Gurugram, India -122002
Email: drhabibhasan@gmail.com
Phone: +91 9999984673

Full name, department, institution, city and country of all co-authors:
1. Dr. Sakthivel Selvaraj, PhD
Director,
Address: Public Health Foundation of India, Plot No. 47, Sector 44, Institutional Area, Gurugram, India -122002

2. Dr. Habib Hasan Farooqui, MD
Associate Professor,
Address: Public Health Foundation of India, Plot No. 47, Sector 44, Institutional Area, Gurugram, India -122002

3. Ms. Aashna Mehta,
Research Associate,
Address: Public Health Foundation of India, Plot No. 47, Sector 44, Institutional Area, Gurugram, India -122002

For peer review only

For peer review only - http://bmjopen.bmj.com/site/about/guidelines.xhtml

Abstract

Does Price Regulation Affect Atorvastatin Sales in India? An Impact Assessment Through Interrupted Time Series Analysis

Objective: The objective of this study was to examine the impact of medicines price regulation (Drug Price Control Order, 2013) on the market share of atorvastatin in the Indian retail market for statins.

Setting: All Indian States, January, 2012 to December, 2015

Design: Quasi experimental - Interrupted Time Series Analysis

Data: Pharmaceutical sales audit dataset from IMS Health (now IQVIA) for the 48 month period from January, 2012 to December, 2015.

Outcome measure : share of atorvastatin (in percentage) in the Indian market for statins in terms of sales volumes.

Results: We observed that the price regulation notification (DPCO,2013) was associated with 0.12 percent ($p<0.001$; 95% CI: 0.06-0.18) increase in the trend of the average monthly market share of atorvastatin (5 mg and 10 mg). After 31 month of price ceilings notification, the average market share of atorvastatin was 3.41 percent higher than would have been expected had the price ceilings not been notified. In sensitivity analysis, with a control, our findings remain robust, we observed a 0.16 percent ($p<0.001$; 95% CI: 0.08-0.24) rise in the trend of average monthly market share of atorvastatin (5 mg and 10 mg) as compared to the change in the control.

Conclusions: Price control as a public intervention did improve the relative sales of atorvastatin in the statin market in India.

Key words: India, price regulation, statin, atorvastatin, interrupted time series

For peer review only - http://bmjopen.bmj.com/site/about/guidelines.xhtml

Article summary

Strengths and limitations of this study

1. To the best of our knowledge this is the first study to report impact of price regulation (DPCO, 2013) on atorvastatin sales in the Indian retail market.
2. The use of quasi-experimental research design –interrupted time series analysis with a control is the key strength of this research.
3. Through the use of nationally representative time series dataset for a 4 year period, the study was able to show long term impact of price regulation.
4. We have not analysed the impact of price regulation on utilization of atorvastatin in the public sector.

For peer review only

For peer review only - http://bmjopen.bmj.com/site/about/guidelines.xhtml

Does Price Regulation Affect Atorvastatin Sales in India? An Impact Assessment Through Interrupted Time Series Analysis

Background

Medicines, vaccines and other medical supplies remain critical and vital elements of the health system.[1] Apart from being part of one of the vital declaration (Alma Ata Declaration) 1978, access to medicines is also one of the six targets of Millennium Development Goal (MDG) 8 to develop a global partnership for development and continues to be a part of the current Sustainable Development Goals (SDGs), 2030.[2] Despite being the "pharmacy of the global south", a significant share of India's population does not have access to essential medicines.[3] A multi-country study utilizing standard WHO methodology has reported that the median availability of a basket of 30 essential medicines in public health facilities ranged from 0-30 percent in six Indian states during 2007.[4] While physical availability of essential medicines in health facilities is a critical indicator, the access framework emphasises the need for examining affordability besides availability. This is particularly important in free market economies where availability of medicines may not be an issue but affordability of medicines is, because it translates into poor or no access for people with low purchasing power. In India, although reported availability of cardiovascular medicines was 81% and 89% in rural and urban private pharmacies respectively, yet only 59% of households were able to afford these cardiovascular medicines.[5] Literature suggests that households in India often spend a large share of their out-of-pocket (OOP) expenditure on healthcare.[6] Studies have also reported impoverishment of households because of OOP medicine expenditures.[5 7] To improve access to healthcare services and reduce financial hardship, national governments resort to various policy instruments, some of them include universal health coverage, publicly funded insurance schemes and pharmaceutical pricing policies.[8] Pharmaceutical pricing policies include policy measures to control medicine prices. These include price control measures like external reference pricing, cost-plus price setting, measures to control add-on costs in the supply chain (such as controlling wholesaler and dispenser mark-ups) and exemption from taxes and/or tariffs.[9] Government's also use price control or limit pharmaceutical profitability as public interventions. Some Organization for Economic Cooperation and Development (OECD) countries for instance, require manufacturers to limit prices in exchange for the subsidies they receive.[10]

India has had progressive drug pricing policies since the year 1979, through the Drug Price Control Orders (DPCO). However, the remit of DPCO had significantly reversed from 1970s when 90% of the market was under price regulation to 2013 by when only about 10% of the market was regulated.[11] The Department of Pharmaceuticals, Ministry of Chemicals and Fertilizers on 7th December, 2012 notified National Pharmaceutical Pricing Policy (NPPP), 2012[12] with the objective "to put in place a regulatory framework for pricing of drugs so as to ensure availability of required medicines - 'essential

For peer review only - http://bmjopen.bmj.com/site/about/guidelines.xhtml

medicines' – at reasonable prices even while providing sufficient opportunity for innovation and competition to support the growth of industry, thereby meeting the goals of employment and shared economic well-being of all." The NPPP 2012 laid down three criteria for price control: (i) regulation of prices based on "essentiality of drugs" (i.e. formulations as listed under the National List of Essential Medicines (NLEM) notified by the Ministry of Health and Family Welfare in 2011)[13], (ii) control of formulation prices only and (iii) market based pricing. The DPCO, 2013 was notified on 15th May, 2013 for the implementation of the NPPP, 2012.

One of the key distinguishing feature of the DPCO, 2013 from the earlier avatars of DPCO include implementation of a new mechanism for price control, called Market Based Pricing (MBP).[14] The central idea of a MBP was to compute the ceiling price of a specific formulation under the NLEM by taking a simple average of the prices to retailers (PTRs) of all brands, with a market share equal to or greater than one percent of the overall market for the said formulation and adding a retailer's margin of 16 percent to obtain the maximum retail price (MRP). In earlier DPCOs of 1979, 1986 and 1995, the mechanism of Cost-plus Based Pricing (CBP) was followed for fixing ceiling prices of formulations by taking into account the raw material cost, conversion cost, packaging material cost and the packing charges. A Maximum Allowable Post Manufacturing Expenses (MAPE), were allowed over and above these costs, to the extent of 100 percent.[15] In addition, unlike earlier price control regimes, the new policy sought to apply price capping only on formulations (finished products) rather than the Active Pharmaceutical Ingredient (API). The total number of medicines under price control as outlined in the NLEM, 2011 were 348 which translated into 628 unique strengths and dosage forms of medicines under DPCO, 2013 (for an indicative calculation of pharmaceutical price cap through DPCO, 2013 see supplementary material). Empirical literature suggests that while direct price control policies are effective in reducing prices and are able to control expenditures, they are unable to reduce medicine expenditures in the long run as manufacturers are able to find ways to increase sales of medicine formulations outside price control.[10] In the Indian context, the selective coverage of the policy led to concerns regarding the shift of sales from price controlled medicines to those outside price control but within the same class of medicines as a result of change in marketing priorities of the companies who would have an incentive to market medicines outside price control.

Statins are the medications of choice for cardiovascular risk reduction (i.e. prevention of heart disease, heart attack and stroke).[16] Previous research on statins use in India has highlighted that inspite of numerous statin products available in Indian retail market, only a fraction of those eligible for a statin appeared to receive the therapy. Authors reported that per capita prescribing rates for statins in India are twenty times lower than those in the United States and Canada.[17] Some of the reasons for low utilization rate of statins in India include limited access to healthcare and affordability. We considered atorvastatin – a class of medicines prescribed to lower blood cholesterol for

For peer review only - http://bmjopen.bmj.com/site/about/guidelines.xhtml

our analysis because India has the highest burden of cardiovascular diseases among developing nations[18] and among all statins, atorvastatin was the only statin under the National List of Essential Medicines, 2011. The price ceilings for atorvastatin (5 mg and 10 mg) were notified on 14th and 21st June, 2013, respectively. The remaining strengths of atorvastatin (20mg and 40mg), other statins such as rosuvastatin, simvastatin, and all combinations of atorvastatin (for example - atorvastatin + acetylsalicilic acid) remain outside price regulation (for detailed information on statin market see tables S1, S2, and S3 in supplementary material).

The effectiveness of pharmaceutical pricing policies depends on the scope and mechanisms around which the regulation is designed. It has been argued that partial price regulation involving one particular drug formulation without considering other equivalent therapeutic formulation may result in switch away from price regulated products to unregulated pharmaceutical products.[19] We hypothesised that on account of new price regulation (DPCO, 2013) there would be a shift in sales of statins from price controlled atorvastatin towards therapeutically equivalent non-price controlled statins. The key objective of this study was to examine the impact of medicine price regulation (Drug Price Control Order, 2013) on the market share of atorvastatin in the Indian retail market for statins through interrupted time series analysis – a quasi-experimental design. To the best of our knowledge, our study is the first attempt from India to generate evidence on the impact of price regulation (DPCO, 2013) on the utilization of atorvastatin used in cardiovascular diseases.

Materials and methods

Data

We used the sales audit dataset from IMS Health (now IQVIA) of the Indian pharmaceutical market for 48 month period from January, 2012 to December, 2015 for this study. IMS Health (now IQVIA) is a for-profit organisation that collects and provides data and information on pharmaceutical market intelligence in over 100 countries around the world . The Indian pharmaceutical sales data is collected from a panel of 5600 stockists across different regions of the country and extrapolated to reflect the private sector sales in the entire country. This comprises the sales made by the stockists to the retailers, hospitals as well as dispensing doctors. The data organizes pharmaceuticals based on anatomical therapeutic classification (ATC) of the European Pharmaceutical Market Research Association (EphMRA). We utilised this information to identify the private market for statins in the country. The data does not capture the public sector utilization of medicines and therefore our analysis pertains only to the impact of the policy on the private sector utilisation of statins in the country.

Outcomes

For peer review only - http://bmjopen.bmj.com/site/about/guidelines.xhtml

Our primary outcome measure was market share (in percentage) in terms of sales volumes. Sales volumes are provided in the sales audit dataset in term of standard units (SUs) which are defined by IMS Health (now IQVIA)) as the smallest dose of formulation which can be one tablet or capsule for oral solids, one vial or ampoule for injectables and so on. We computed the monthly share of atorvastatin (5 mg and 10 mg), the statin under the NLEM and hence within the ambit of price regulation, in terms of sales volume in the statins market for the time period under study. Medicine sales was taken as a proxy for utilization of the specific formulation for the purpose of our analysis.

Research design

We used interrupted time series, a quasi-experimental research design to capture the impact of price ceiling on utilization of atorvastatin (5 mg and 10 mg were notified in under DPCO in June, 2013).[5] Rosuvastatin (all strengths), a statin outside price regulation was used as the control to further strengthen our research design.

Statistical analysis

The intervention under study is the notification of price ceilings for atorvastatin (5 mg and 10 mg). Specifically, the price ceiling for atorvastatin 5 mg and atorvastatin 10 mg were notified on 14th June, 2013 and 21st June, 2013 respectively. The 48 month period under study was distributed into two segments. The pre-intervention 17 month period from January, 2012 to May, 2013 and the post-intervention period of 31 months starting June, 2013 to December, 2015. We did not take into consideration the period after December 2015 as a new NLEM was notified in this period. Based on the new NLEM, 2015 price ceilings were subsequently notified for atorvastatin 10 mg, 20 mg and 40 mg in 2016.

We used segmented linear regression analysis[20] to detect the pre-intervention trends, post-intervention level changes, and changes in post-intervention trends relative to the pre-intervention trends in the use of atorvastatin (5mg and 10mg). The dependent variable (Y_t) was 'market share' of atorvastatin (5 mg and 10 mg) in terms of sales volumes while 'time' appeared as an independent variable. We fitted a least square regression line to the two segments of the continuous variable time. In addition, we introduced two binary variables to estimate immediate level change after the intervention (variable name: intervention) as well as trend change (variable name: time after intervention) in the market share of regulated atorvastatin (see equation 1 below). The variable 'intervention' was 0 for the pre-intervention period and 1 for the post-intervention period starting June, 2013 (model 1). Time after intervention was a continuous variable starting June, 2013.[6]

$$Y_t = \alpha + \beta_1 \text{time}_t + \beta_2 \text{ intervention}_t + \beta_3 \text{ time after intervention}_t + \varepsilon_t \quad \text{........equation 1}$$

For peer review only - http://bmjopen.bmj.com/site/about/guidelines.xhtml

The segmented regression helped us statistically estimate the change in the intercept and the slope coefficients between the pre and post-intervention period. We checked the model for autocorrelation with the help of Durbin-Watson statistic, autocorrelation (ac) and partial autocorrelation (pac) estimates and plots of the residuals. We did not detect autocorrelation in our models (see figures 1a, 1b, 1c and 2a, 2b, 2c in supplementary material for details). Seasonality is unlikely to influence the outcome measure as the group of medicines under study are intended for a chronic condition and meant to be consumed throughout the lifetime of the patients.

In addtition, we ran an alternate model (model 2) wherein we excluded the adjustment period of two months from June to August 2013 since manufacturers were provided a maximum of 45 days to adjust their maximum retail prices in accordance with the notified ceiling price. Inspecting the data also revealed that this period saw a steep decline in the share of price regulated atorvastatin in the statin market which suggests that the suppliers were withdrawing the stocks from the market to re-label the medicine packs with the revised MRPs. However, we do not have a way of knowing which manufacturers or how many of them adjusted the ceiling prices in a weeks' time, two weeks' time, three weeks' time and so on. Further, we do not believe that the availability of this information would have impacted our results significantly or that the implementation period was long enough to affect our results. A counterfactual was introduced into both the models, i.e. trend in utilization of atorvastatin in the post-intervention period had the price ceilings not been notified. It is predicted that in the absence of price ceiling, pre-intervention trend in utilization would have remained constant in the post-intervention period. We computed the difference between the predicted values of the market share of atorvastatin for the actual as well as the counterfactual scenarios to get the estimate of the absolute policy effect.

Finally, a control was introduced in the model (model 3) to strengthen our study design. The marker share of rosuvastatin, a statin completely outside the ambit of price regulation was used as control. Rosuvastatin was chosen as control instead of other statins such as simvastatin, because rosuvastatin is the second highest selling plain formulation in both value and volume terms after atorvastatin. The intention was to control for time varying confounders and other interventions that may have affected the outcome of interest. We computed the difference in the respective market shares of atorvastatin (5 mg and 10 mg) under price regulation and rosuvastatin, our comparison group. We then ran the above described interrupted time series model with the difference in the market share as our new dependent variable. This method had the advantage of simultaneously controlling the pre-intervention differences in the utilization level and trend between the two statins. Once again, two separate models were run – with and without the adjustment period (model 3). We, additionally ran our interrupted time series model on total sales volumes (in SUs) per capita instead of market share for atorvastatin (5 mg and 10mg) as well statins as a whole to check if the policy has an impact on the sales volumes of the entire statins market.

For peer review only - http://bmjopen.bmj.com/site/about/guidelines.xhtml

Patient and Public Involvement

Patients or the public weren't involved in this study, which is based on secondary data.

Results

Our analysis suggest that statin market which was worth INR 22.90 billion in 2015, accounted for 2.25 percent of the Indian pharmaceutical market in terms of sales value and 0.63 percent of the market in terms of sales volume(Table 1).The market value and volume of statins increased between 2012 and 2015 both in absolute terms as well as in terms of relative share in the overall pharmaceutical market. It is interesting to note that for the time period 2012 to 2015, the share of fixed dose combinations of statins have been rising and that of plain formulation has been falling in terms of both sales values and volumes.

The contribution of atorvastatin in overall statin market, was 44.9 % and 48.9 % in volume and value terms in the year 2012, respectively which went down to 35.7% and 38.2% of the overall statin market by the year 2015 (Table 2). We observed that the market share of regulated atorvastatin (5mg and 10 mg) in the statins market has also been falling in terms of both sales values and volumes for the period 2012 to 2015. For the same period, the market share of another statin, rosuvastatin has been increasing in both value and volume terms. The difference between the market shares of both the statins in terms of sales volume has been narrowing over the study period. In terms of sales values however, the difference between the two market shares turned negative in 2013 and the gap has been widening up to 2015.

Table 1: Statins in the Indian Pharmaceutical Market

**Statins Market**	**Volumes in billion SUs (%)**				**Values in INR billions (%)**			
	**2012**	**2013**	**2014**	**2015**	**2012**	**2013**	**2014**	**2015**
Overall Statins Market (share in the Indian pharmaceutical market)	2.50 (0.50)	2.81 (0.56)	3.15 (0.60)	3.56 (0.63)	15.47 (2.16)	16.92 (2.14)	19.41 (2.20)	22.90 (2.25)
FDCs (share in statins market)	0.94 (37.56)	1.14 (40.44)	1.31 (41.76)	1.55 (43.40)	4.56 (29.46)	5.21 (30.79)	6.48 (33.40)	7.92 (34.56)
Plain	1.56	1.67	1.83	2.02	10.91	11.71	12.93	14.99

For peer review only - http://bmjopen.bmj.com/site/about/guidelines.xhtml

formulations (share in statins market)	(62.44)	(59.56)	(58.24)	(56.60)	(70.54)	(69.21)	(66.60)	(65.44)
Total Market	504357.87	504757.49	523098.93	563619.26	715163.62	788943.16	882423.34	1015741.19

Note: SUs refer to Standard Units; INR refers to Indian National Rupee

Table 2: Share of atorvastatin 5 mg and 10 mg, other strengths of atorvastatin and rosuvastain in the statins market.

	Sales volumes (%)				Sales values (%)			
	**2012**	**2013**	**2014**	**2015**	**2012**	**2013**	**2014**	**2015**
**(A)** Atorvastatin (5mg & 10mg) (%)	32.34	28.87	26.29	24.1	25.84	22.4	17.01	15.3
**(B)** Atorvastatin (Other Strengths) (%)	12.63	11.88	11.81	11.64	23.09	22.57	23.19	22.93
**(C=A+B)** Atorvastatin (all strengths)	44.97	40.75	38.1	35.73	48.93	44.96	40.2	38.23
**(D)** Rosuvastatin (all strength) (%)	15.79	17.53	19.15	20.12	19.65	22.66	25.16	26.27
**(E=A-D)** Difference between Atorvastatin (5mg & 10mg) & Rosuvastatin (%)	16.55	11.34	7.14	3.98	6.19	-0.26	-8.15	-10.97

Our results from segmented regression analysis (model 1) suggest that post intervention, there was an immediate reduction in the average monthly market share of atorvastatin by 0.25 percent but this change in level was insignificant. This was followed by a significant increase in the trend of 0.12 percent ($p<0.001$)) per month. Figure 1 (a) demonstrates fitted values (solid line) of the market share of atorvastatin in the pre and post-intervention period. Additionally, the figure also captures the counterfactual fitted values of market share (dotted line). The counterfactual represents the scenario had the price ceilings for atorvastatin not been notified during the period under study. The vertical line in the figure at June 2013 represents the intervention period which in our case is the month in which price ceilings were notified for atorvastatin (5 mg and 10 mg). It is evident from figure 1 (a) and table 3 that the effect was negative for the first couple of months. This was followed by a sustained positive effect for the remaining duration. In December, 2015, the 31st month after the

For peer review only - http://bmjopen.bmj.com/site/about/guidelines.xhtml

notification of price ceilings, the average market share of atorvastatin was 3.41 percent higher than would have been expected had the price ceilings not been notified.

We ran another model to check whether the implementation period impacted our results significantly. In model 2, we excluded the period from June 2013 to August 2013. Upon excluding the two-month period, we did not observe a significant modification in our results (see figure 1(b)). The post-intervention level change continued to be negative and insignificant, although the magnitude increased by 0.23 and the post-intervention trend change slightly reduced by 0.01 but continued to be significant (p<0.001). In December 2015, the 31st month after the notification of price ceilings, the average market share of atorvastatin was 2.28 percent higher than would have been expected had the price ceilings not been notified (see table S4 in supplementary material for absolute policy effect from the intervention point June, 2013 up to December, 2015). Excluding the implementation period did not alter our results significantly.

Table 3: Segmented Regression analysis results for drug utilization (atorvastatin 5mg and 10 mg)

	**Model 1**			**Model 2**		
**Variable**	**Coefficients**	**95% conf. interval**		**Coefficients**	**95% conf. interval**	
**Time**	-0.31 (***)	-0.36	-0.25	-0.31 (***)	-0.36	-0.25
**Intervention (level change)**	-0.25	-0.9	0.41	-0.48	-1.12	0.15
**Time after intervention (trend change)**	0.12 (***)	0.06	0.18	0.11 (***)	0.05	0.17
**_Const**	34.34 (***)	33.78	34.9	34.34 (***)	33.8	34.88
**No. of observations**	48 (pre-intervention: 17 and post-intervention: 31)			45 (pre-intervention: 17 and post-intervention: 28)		
**R-squared**	0.9828			0.9763		

Note: *** refers p<0.001 and ** refers to p<0.05

To increase the robustness of our analysis, we introduced a control (model 3)– another statin - rosuvastatin (all strengths) which was outside the ambit of price regulation. Table 4 and figure 2 presents result of the segmented regression analysis with the difference in the market shares of atorvastatin (5 mg and 10 mg) and rosuvastatin as the dependent variable. We observed a 0.69 percent (p>0.05) immediate post-intervention drop in the average monthly difference in the market share of atorvastatin (5 mg and 10 mg) followed by a 0.16 percent (p<0.001) rise in the monthly trend as

For peer review only - http://bmjopen.bmj.com/site/about/guidelines.xhtml

compared to the change in the control, rosuvastatin. Our results after factoring in the control reiterate robustness of our findings i.e. the average monthly trend of the difference in the market share of atorvastatin (5mg and 10 mg) is positive in the post-intervention period. Therefore our null hypotheses – that price regulation would lead to a switch away from price regulated atorvastatin (5 mg and 10 mg) towards statins outside price ceiling stands rejected.

In summary, the DPCO 2013 regulation led to a shift towards the price regulated atorvastatin (5 mg and 10 mg) from other statins which were not under price regulation. Finally, we did not observe any effect of the policy on the per capita sales volumes (in SUs) of either atorvastatin (5mg and 10mg) or statins as a whole. The policy did not significantly impact the sales of regulated atorvastatin in absolute terms or statins as a whole. The impact was relative and therefore can be interpreted as a switch towards the regulated atorvastatin.

Table 4: Segmented Regression analysis results for drug utilization (atorvastatin 5mg and 10mg with control)

	**Model 3**		
**Variable**	**Coefficients**	**95% conf. interval**	
**Time**	-0.45 (***)	-0.52	-0.37
**Intervention (level change)**	-0.69	-1.54	0.16
**Time after intervention (trend change)**	0.16 (***)	0.08	0.24
**_Const**	19.49 (***)	18.76	20.22
**No. of observations**	48 (pre-intervention: 17 and post-intervention: 31)		
**R-squared**	0.9808		

Note: *** refers p<0.001; ** refers to p<0.05

Discussion

We have generated robust evidence on the impact of price regulation policy (DPCO, 2013) on the sales of atorvastatin (5mg and 10 mg) through the use of interrupted time series analysis –a quasi-experimental design. The use of interrupted time series analysis to measure the policy impact by comparing pre-intervention versus post-intervention trends has increased in recent past especially to assess impact of health related interventions.[21-27] Our research design, analytic approach and reporting conform to well establish methodological standards.[28 29]

We observed that the baseline trend for average monthly atorvastatin sales was declining during the study period (January 2012 to December 2015). Some of the reasons for such trend includes prescription preferences, availability of therapeutically

For peer review only - http://bmjopen.bmj.com/site/about/guidelines.xhtml

substitutable products and marketing practices of manufactures. However, the sales of the price controlled formulation of atorvastatin (5mg and 10mg) relative to other statins increased after the ceiling price notification in June 2013. Further, after an initial downward shift in sales, which could have resulted from the recall of existing packs of atorvastatin from the retail market for repackaging and reprinting the new maximum retail prices (MRPs)[30] as mandated in DPCO 2013, there was a sustained positive effect of regulation as reflected in increasing monthly sales. This sustained positive effect may have resulted primarily because of price reductions leading to increased untilization of the price regulated atorvastatin (5mg and 10mg) in comparison to other statins on account of increased affordability. Similarly, Damiani et al. evaluated national and regional cost containment measures (i.e. revised national reimbursement criteria and regional co-payment) on statins use in Italy using interupted time series analysis and reported that the regional copayment was associated with a small increase in trend of statin use, whereas restriction to reimbursement interventions was associated with an immediate drop and a decrease in trend of statin use, highlighting that public policies directed towards cost containment may impact statin utilization.[31]

Empirical literature suggests that direct price control policies are usually unable to reduce medicine expenditures as manufacturers are able to find ways to increase sales of medicine formulations outside regulation.[10] Recent research on price control of anti-hypertensive medicines from Korea reported some unintended effect of the policy i.e. drug price reduction resulted in drug over-utilisation and use of prohibited combinations. Also, the utilisation of drugs, which was not affected by the drug price reduction, increased by 12.3%.[32] Studies from Norway and Finland have reported that policy interventions directed towards physicians to prescribe less expensive statins and restricting reimbursement of expensive statins have resulted in increase in the consumption of statins and, simultaneously, a decrease in the expenditure.[33 34] World Health Organization guidelines on pharmaceutical pricing policies also suggest that at any given point of time national governments should employ a judicious mix of different policy instruments to control medicine prices and expenditure.[9]

Our study has certain limitations. We have evaluated the impact of price regulation on only statins market, our findings are not representative for other medicines or formulations under price control. In addition, after the announcement of National List of Essential Medicines (NLEM), 2015, few more strengths of atorvastatin (20mg and 40mg) have been brought under price control through Drugs (Price Control) Amendment Order, 2016.[35] To ascertain the effect of increased coverage of price regulation, another segmented regression analysis should be conducted after sufficient time period has lapsed after implementation of Drugs (Price Control) Amendment Order, 2016. More research is needed to measure the impact of price regulation in other markets on the basis of on other medicines which are part of NLEM 2015. We have also not evaluated the effect of increased utilization of atorvastatin on health outcomes. We have not analysed the impact of price regulation on atorvastatin utilization in the public

For peer review only - http://bmjopen.bmj.com/site/about/guidelines.xhtml

sector. Although, the price regulation policy has no intended effect on public sector utilization, since medicine procurement in public sector is handled through medical services corporations which procure medicines through tender based system.

Conclusions

The evidence emerging from our study indicates that the medicine price regulation (DPCO 2013) had sustained positive impact on atorvastatin utilization in comparison to other statins as reflected in increasing average monthly sales in the post-intervention period. However, the policy impact was relative, interpreted as a switch towards the regulated atorvastatin. It should be noted that the impact of price regualtion may vary across other medicines or formulation on account of competition and concentration in each therapeutic segment, manufacturer's marketing practices, physician's prescribing behavior and consumer behavior. In addition, policy makers should also consider unintended effects of the policy, such as manufacturers response to reduced prices and consequently reduced profitability of the formulations. To enhance affordability of medicines, the price regulation should be accompanied with other regulatory reforms and policy measures, universal access to essential medicines could be one of them.

Contributions: SS, HHF, and AM conceived the idea, designed the analysis, conducted data analysis and wrote the first draft of the paper. SS, HHF, and AM conducted the literature review and the interpretation of the results. SS, HHF, and AM revised and edited the manuscript to its final stages. All the authors approved the final manuscript version.

Acknowledgments: The authors would like to thank Dr. Anup Karan (Asscoiate Professor, Indian Institute of Public Health, Public Health Foundation of India) for his critical and constructive comments on the methodology.

Competing Interests: We declare no conflict of interest.

Funding: Habib Hasan Farooqui is supported by a Wellcome Trust Capacity Strengthening Strategic Award (084754/Z/08/A) to the Public Health Foundation of India and a consortium of UK universities. The funders had no role in study design, data collection and analysis, decision to publish, or preparation of the manuscript.

Data sharing: The IMS health data is available on request, at the approval of IMS Health.

Ethical approval: Ethical approval for this study was not needed. The study used only anonymised data from secondary sources.

Reference:

For peer review only - http://bmjopen.bmj.com/site/about/guidelines.xhtml

For peer review only

1. Bigdeli M, Jacobs B, Tomson G, et al. Access to medicines from a health system perspective. *Health policy and planning* 2013;28(7):692-704. doi: 10.1093/heapol/czs108
2. Gotham D, Onarheim KH, Barber MJ. How the MDGs gave up on measuring access to medicines. *The Lancet Global health* 2016;4(5):e296-7. doi: 10.1016/S2214-109X(16)00066-8
3. WHO. THE WORLD MEDICINES SITUATION. Geneva: World Health Organization, 2004.
4. Cameron A, Ewen M, Ross-Degnan D, et al. Medicine prices, availability, and affordability in 36 developing and middle-income countries: a secondary analysis. *Lancet* 2009;373(9659):240-9. doi: 10.1016/S0140-6736(08)61762-6
5. Khatib R, McKee M, Shannon H, et al. Availability and affordability of cardiovascular disease medicines and their effect on use in high-income, middle-income, and low-income countries: an analysis of the PURE study data. *Lancet* 2016;387(10013):61-9. doi: 10.1016/S0140-6736(15)00469-9
6. Karan A, Selvaraj S, Mahal A. Moving to universal coverage? Trends in the burden of out-of-pocket payments for health care across social groups in India, 1999-2000 to 2011-12. *PloS one* 2014;9(8):e105162. doi: 10.1371/journal.pone.0105162
7. Rao KD, Bhatnagar A, Murphy A. Socio-economic inequalities in the financing of cardiovascular & diabetes inpatient treatment in India. *Indian J Med Res* 2011;133:57-63.
8. Veronika J Wirtz HVH, Andrew L Gray, Maryam Bigdeli, Cornelis P de Joncheere, Margaret A Ewen, et. al. Essential medicines for universal health coverage. *Lancet* 2016;389:403-76.
9. WHO. WHO Guideline on Country Pharmaceutical Pricing Policies. Geneva: World Health Organization, 2013.
10. MSH. Pharmaceutical Pricing Policy: Management Sciences for Health, 2012.
11. Sakthivel Selvaraj HHF. Legitimising Unaffordable Medicine Prices? Draft Drug Price Policy 2011. *Economic and Political Weekly* 2012;Vol. 47,(Issue No. 46,)
12. National Pharmaceutical Pricing Policy 2012. India: Government of India, 2012.
13. MOHFW. National List of Essential Medicines (NLEM) 2011. New Delhi: Ministry of Health and Family Welfare, 2011.
14. India Go. Drug Price Control Order 2013. In: Department of Pharmaceuticals MoCaF, ed. India, 2013.
15. India Go. Drug Price Control Order 1995. In: Department of Pharmaceuticals MoCaF, ed. India, 1995.
16. Niteesh K. Choudhry SD, William H. Shrank, Jennifer M. Polinski, Christina E. Stark, Rajeev Gupta, Dorairaj Prabhakaran, Gregory Brill and Prabhat Jha. Despite Increased Use And Sales Of Statins In India, Per Capita Prescription Rates Remain Far Below High-Income Countries. *Health Affairs,* 2014;33(2):273-82.
17. Choudhry NK, Dugani S, Shrank WH, et al. Despite increased use and sales of statins in India, per capita prescription rates remain far below high-income countries. *Health Aff (Millwood)* 2014;33(2):273-82. doi: 10.1377/hlthaff.2013.0388
18. Patel V CS, Chisholm D, Ebrahim S, Gopalakrishna G, Mathers C, et al. . Chronic diseases and injuries in India. . *Lancet* 2011;377(9763):413-28.
19. Ajay Bhaskarabhatla CC, Priyatam Anurag and Enrico Pennings. Mitigating regulatory impact: the case of partial price controls on metformin in India. *Health policy and planning* 2016

For peer review only - http://bmjopen.bmj.com/site/about/guidelines.xhtml

20. Singal GL, Nanda A, Kotwani A. A comparative evaluation of price and quality of some branded versus branded-generic medicines of the same manufacturer in India. *Indian journal of pharmacology* 2011;43(2):131-6. doi: 10.4103/0253-7613.77344
21. Yoo K-B LS, Park S, et al. . Effects of drug price reduction and prescribing restrictions on expenditures and utilisation of antihypertensive drugs in Korea. . *BMJ Open* 2015;5
22. Garabedian LF R-DD, Ratanawijitrasin S, et al. Impact of universal health insurance coverage in Thailand on sales and market share of medicines for non-communicable diseases:ban interrupted time series study. . *BMJ Open* 2012;2
23. Leopold C M-TA, Seyfang L, Vogler S, Joncheere KD, Laing OR, Leufkens H. . Impact of External Price Referencing on Medicine Prices – a Price Comparison among 14 European countries. . *Southern Med Review* 2012;5(2):34-41.
24. Gianfranco Damiani BF, Angela Anselmi, Caterina Bianca Neve Aurora Bianchi, Giulia Silvestrini, et. al. The impact of Regional co-payment and National reimbursement criteria on statins use in Italy: an interrupted time-series analysis. *BMC health services research* 2014;14(6)
25. Evangelos Kontopantelis TD, David A Springate,Iain Buchan,David Reeves. Regression based quasi-experimental approach when randomisation is not an option: interrupted time series analysis. *Bmj* 2015;350
26. Lagarde M. How to do (or not to do) . . . Assessing the impact of a policy change with routine longitudinal data. *Health policy and planning* 2011
27. James Lopez Bernal SC, Antonio Gasparrini. Interrupted time series regression for the evaluation of public health interventions: a tutorial. *International Journal of Epidemiology,* 2016
28. Wagner AK, Soumerai SB, Zhang F, et al. Segmented regression analysis of interrupted time series studies in medication use research. *J Clin Pharm Ther* 2002;27(4):299-309.
29. Racquel Jandoca AMB, Muhammad Mamdania,b,c, Linda E. L evesqued,, Cadarettea SM. Interrupted time series analysis in drug utilization research is increasing: systematic review and recommendations. *Journal of Clinical Epidemiology* 2015;68:950-56.
30. NPPA. Compendium of notified ceiling prices of schedules drug - 2015: National Pharmaceutical Pricing Authority, 2015.
31. Damiani G, Federico B, Anselmi A, et al. The impact of regional co-payment and national reimbursement criteria on statins use in Italy: an interrupted time-series analysis. *BMC Health Serv Res* 2014;14:6. doi: 10.1186/1472-6963-14-6
32. Kwon HY, Hong JM, Godman B, et al. Price cuts and drug spending in South Korea: the case of antihyperlipidemic agents. *Health policy* 2013;112(3):217-26. doi: 10.1016/j.healthpol.2013.08.011
33. Sakshaug S FK, Karlstad Ø, Rønning M, Skurtveit S. Switching statins in Norway after new reimbursement policy: a nationwide prescription study. *Br J Clin Pharmacol* 2007;64(4):476-81.
34. Martikainen JE SL, Korhonen MJ, Enlund H, Helin-Salmivaara A. Impact of restricted reimbursement on the use of statins in Finland: a register-based study. *Med Care* 2010;48(9):761-6.
35. Bureau PI. Ceiling prices of 464 formulation fixed after announcement of NLEM, 2015 and Revised Schedule-I, resulting in savings of Rs 2288 crore for

For peer review only - http://bmjopen.bmj.com/site/about/guidelines.xhtml

consumers 2016 [Available from: http://pib.nic.in/newsite/PrintRelease.aspx?relid=151146 accessed June 2017.

Figure legends:

Figure 1: Fitted values of the market share of atorvastatin 5 mg and 10 mg, Model 1 and Model 2 - actual and counterfactual
Note: ms refers to the market share of atorvastatin (5mg and 10mg)

Figure 2: Fitted values of the difference in the market share of atorvastatin (5 mg, 10 mg) and Rosuvastatin (all strengths) - actual and counterfactual
Note: ms_diff refers to the difference between the market share of atorvastatin (5mg and 10mg) and rosuvatstain (all strength); ms_ator refers to the market share of atorvastatin (5mg and 10mg); and ms_rosuv refers to the market share of rosuvatstain (all strength)

For peer review only

For peer review only - http://bmjopen.bmj.com/site/about/guidelines.xhtml

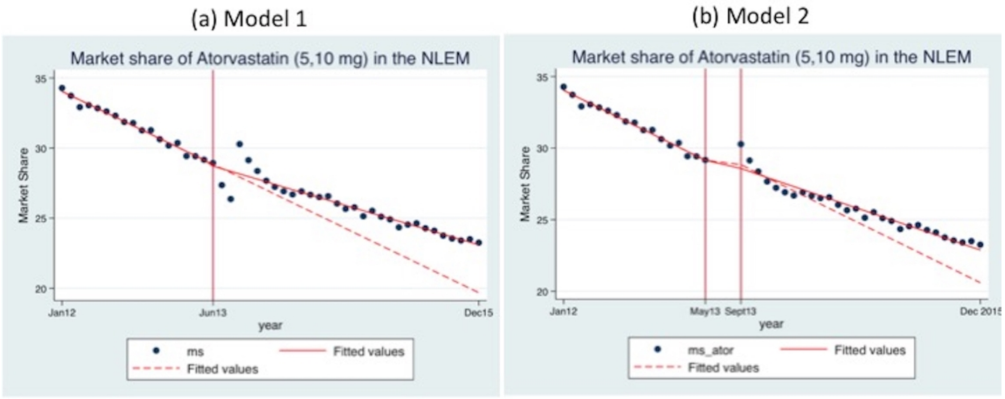

Caption : Figure 1: Fitted values of the market share of atorvastatin 5 mg and 10 mg, Model 1 and Model 2 - actual and counterfactual
Note: ms refers to the market share of atorvastatin (5mg and 10mg)

254x190mm (300 x 300 DPI)

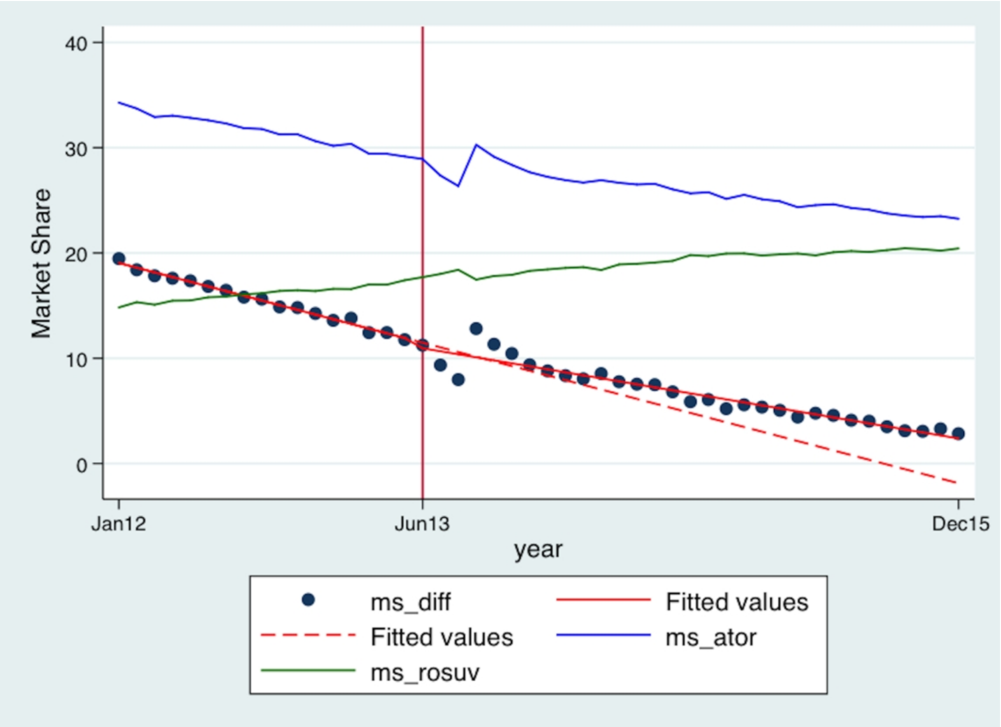

Figure 2: Fitted values of the difference in the market share of atorvastatin (5 mg, 10 mg) and Rosuvastatin (all strengths) - actual and counterfactual
Note: ms_diff refers to the difference between the market share of atorvastatin (5mg and 10mg) and rosuvatstain (all strength); ms_ator refers to the market share of atorvastatin (5mg and 10mg); and ms_rosuv refers to the market share of rosuvatstain (all strength)

139x101mm (300 x 300 DPI)

Supplementary materials

Indicative calculation of pharmaceutical price cap :

The ceiling prices in DPCO, 2013 were computed using the Market Based Pricing formula[i] as under:

First the Average Price to Retailer, P(s) was computed as under:

P(s) = (Sum of prices to retailer of all the brands and generic versions of the medicine having market share more than or equal to one percent of the total market turnover on the basis of moving annual turnover of that medicine) / (Total number of such brands and generic versions of the medicine having market share more than or equal to one percent of total market turnover on the basis of moving annual turnover for that medicine.)

Then ceiling price was obtained by adding the retailer's margin of 16 percent.

P(c) = P(s).(1+M/100), where
P(s) = Average Price to Retailer for the same strength and dosage of the medicine as calculated in step1 above.
M = % Margin to retailer and its value=16

An indicative calculation of pharmaceutical price cap is presented in Table S1, with an illustration of Atorvastatin market, involving the strength of 5mg and 10mg tablet, underlying the period before and after price fixation. During the period under consideration, there were a total of 33 packs sold in the market involving Atorvastatin 5 mg and 100 plus packs of Atorvastatin 10 mg. Going by principle of only packs with more than or equal to one percent market share in that category, only half and one-fourth of the market gets included in price ceiling calculation for Atorvastatin 5 mg and Atorvastatin 10 mg respectively.

Table S1 : Price to Retailers (PTR) for Atorvastatin

Price Indicators	All packs				Packs with market share>1%			
	2012	2013	2014	2015	2012	2013	2014	2015
Strength: 5mg								
No. of packs	33	33	33	35	18	17	17	16
Median PTR	3.21	3.25	3.09	2.97	3.65	3.71	3.22	3.31
Average PTR	2.94	2.95	2.69	2.75	3.34	3.36	3.02	3.13
Lowest PTR	0.69	0.70	0.79	0.84	1.22	1.33	1.39	1.41
Highest PTR	5.10	4.68	4.96	4.96	5.10	4.68	4.96	4.96
Strength: 10mg								
No. of packs	100	106	109	111	23	25	22	23
Median PTR	4.7	4.9	4.79	4.65	6.41	5.99	4.88	5.11
Average PTR	4.63	4.61	4.28	4.09	5.73	5.15	4.33	4.56

For peer review only

Lowest PTR	0.91	0.95	1.05	0.76	2.21	1.73	1.59	1.65
Highest PTR	8.72	10.47	12.14	10.48	7.98	7.26	5.54	5.67

Several dimensions of price parameters are worth observing from Table S1. In particular, it may be observed that the lowest price-to-retailer (PTR) is markedly lower when all packs are considered, both under 5mg and 10mg. Under price ceiling scenario involving only players with one percent market share, not only the lowest PTR is significantly but median and average PTR involving more than one percent market share is also considerably higher than PTR for all packs.In respect to price ceiling, it is evident that the drug price (measured by price-to-retailer) of Atorvastatin of all packs were significantly lower than packs with one percent market share, whether measured by average or median PTR.

Table S2: Statins in the Indian Pharmaceutical Market

Statins Market	Volumes in billion Sus (%)				Values in INR billions (%)			
	2012	2013	2014	2015	2012	2013	2014	2015
Oveall Statin Market Share	2.50 (0.50)	2.81 (0.56)	3.15 (0.60)	3.56 (0.63)	15.47 (2.16)	16.92 (2.14)	19.41 (2.20)	22.90 (2.25)
FDCs (share in statins market)	0.94 (37.56)	1.14 (40.44)	1.31 (41.76)	1.55 (43.40)	4.56 (29.46)	5.21 (30.79)	6.48 (33.40)	7.92 (34.56)
Plain formulations (share in statins market)	1.56 (62.44)	1.67 (59.56)	1.83 (58.24)	2.02 (56.60)	10.91 (70.54)	11.71 (69.21)	12.93 (66.60)	14.99 (65.44)
Total Market	504357.87	504757.49	523098.93	563619.26	715163.62	788943.16	882423.34	1015741.19

Table S3: Top 5 formulations in the Statins market in terms of values and volumes in the year 2012

	Share in terms of Volumes (%)				Share in terms of Values (%)			
Statins	2012	2013	2014	2015	2012	2013	2014	2015
Atorvastatin Calcium Salt	44.97	40.75	38.10	35.73	48.93	44.96	40.20	38.23
Acetylsalicylic Acid + Atorvastatin Calcium Salt	19.79	20.78	20.86	21.86	5.84	5.55	6.63	6.81
Rosuvastatin Calcium Salt	15.79	17.53	19.15	20.12	19.65	22.66	25.16	26.27
Atorvastatin Calcium Salt + Fenofibrate	6.01	5.45	5.04	4.50	8.55	7.73	7.25	6.37

For peer review only

Fenofibrate + Rosuvastatin Calcium Salt	2.90	3.64	3.97	4.25	4.31	6.13	6.72	7.17
Total share of top 5 statins	89.46	88.14	87.13	86.47	87.28	87.04	85.95	84.84
Other statins	10.54	11.86	12.87	13.53	12.72	12.96	14.05	15.16

Table S4: Absolute policy effect starting the intervention period (model 1 and 2)

	Model 1			Model 2		
Month	Predicted values_actual	Predicted values_counterfactual	Absolute policy effect	Predicted values_actual	Predicted values_counterfactual	Absolute policy effect
Jun-13	28.72	28.84	-0.13			
Jul-13	28.53	28.54	-0.01			
Aug-13	28.34	28.23	0.11	28.47	28.84	-0.37
Sep-13	28.15	27.93	0.23	28.28	28.54	-0.26
Oct-13	27.97	27.62	0.34	28.08	28.23	-0.15
Nov-13	27.78	27.32	0.46	27.89	27.93	-0.04
Dec-13	27.59	27.01	0.58	27.69	27.62	0.07
Jan-14	27.40	26.71	0.7	27.50	27.32	0.18
Feb-14	27.22	26.40	0.82	27.31	27.01	0.29
Mar-14	27.03	26.10	0.93	27.11	26.71	0.40
Apr-14	26.84	25.79	1.05	26.92	26.40	0.52
May-14	26.65	25.49	1.17	26.72	26.10	0.63
Jun-14	26.47	25.18	1.29	26.53	25.79	0.74
Jul-14	26.28	24.88	1.41	26.33	25.49	0.85
Aug-14	26.09	24.57	1.52	26.14	25.18	0.96
Sep-14	25.91	24.26	1.64	25.95	24.88	1.07

For peer review only

Oct-14	25.72	23.96	1.76	25.75	24.57	1.18
Nov-14	25.53	23.65	1.88	25.56	24.26	1.29
Dec-14	25.34	23.35	2	25.36	23.96	1.40
Jan-15	25.16	23.04	2.11	25.17	23.65	1.51
Feb-15	24.97	22.74	2.23	24.97	23.35	1.63
Mar-15	24.78	22.43	2.35	24.78	23.04	1.74
Apr-15	24.59	22.13	2.47	24.59	22.74	1.85
May-15	24.41	21.82	2.59	24.39	22.43	1.96
Jun-15	24.22	21.52	2.7	24.20	22.13	2.07
Jul-15	24.03	21.21	2.82	24.00	21.82	2.18
Aug-15	23.85	20.91	2.94	23.81	21.52	2.29
Sep-15	23.66	20.60	3.06	23.61	21.21	2.40
Oct-15	23.47	20.30	3.18	23.42	20.91	2.51
Nov-15	23.28	19.99	3.29	23.23	20.60	2.63
Dec-15	23.10	19.68	3.41	23.03	20.30	2.74

For peer review only

Tests for Autocorrelation in model 1

Figure 1a.

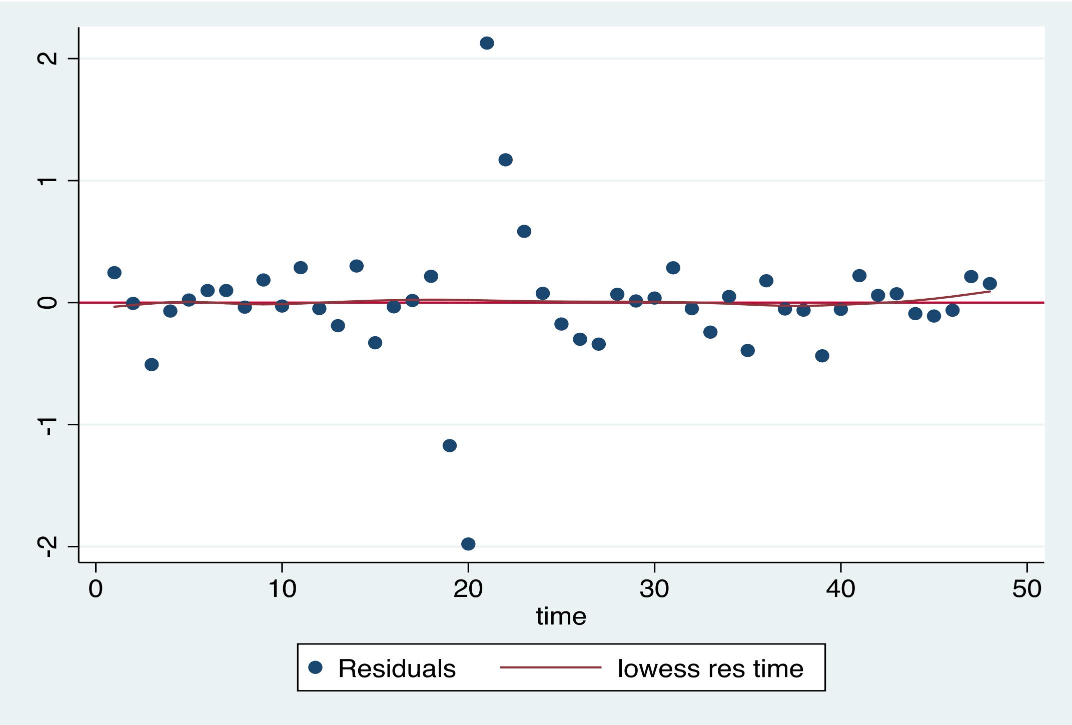

Figure 1b.

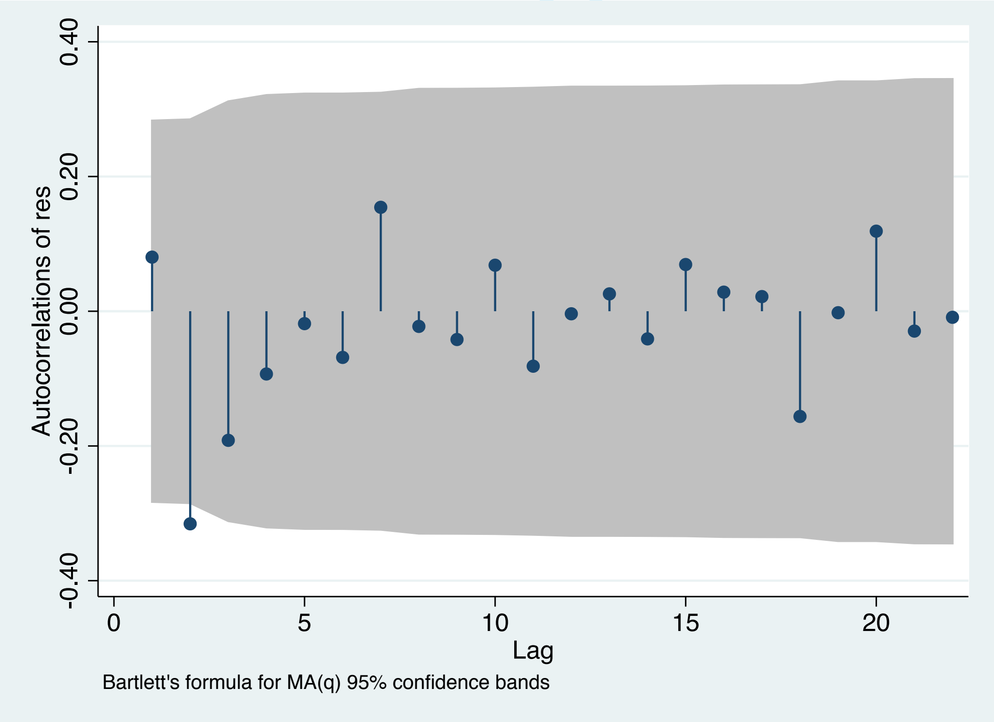

Figure 1c.

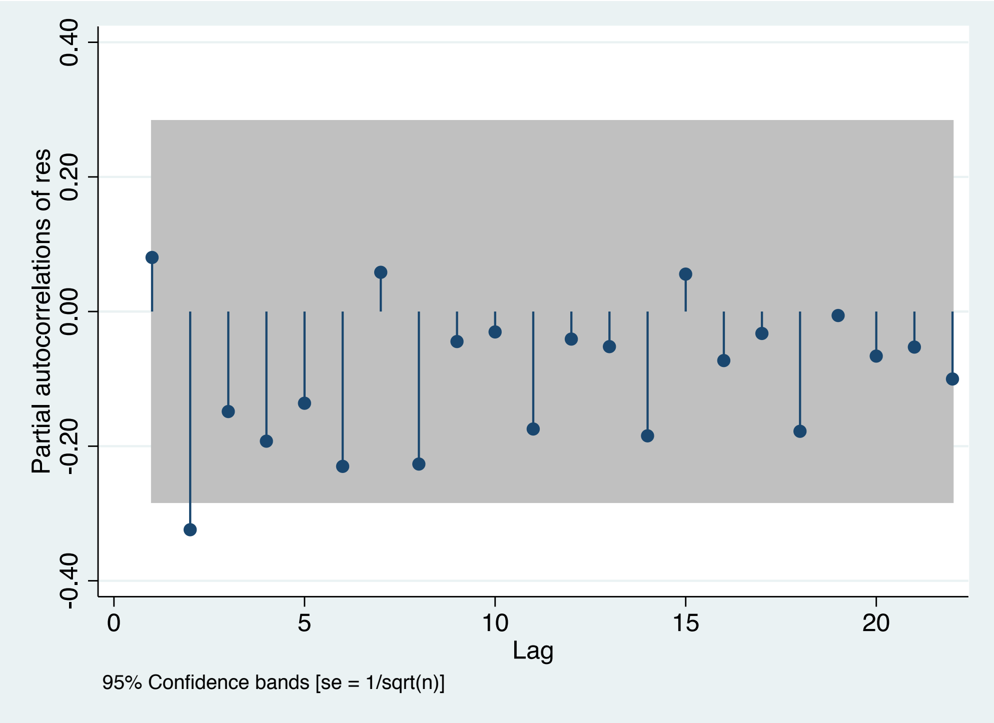

Durbin-Watson d-statistic(4, 48) = 1.833076

Tests for Autocorrelation in model 2

Figure 2a.

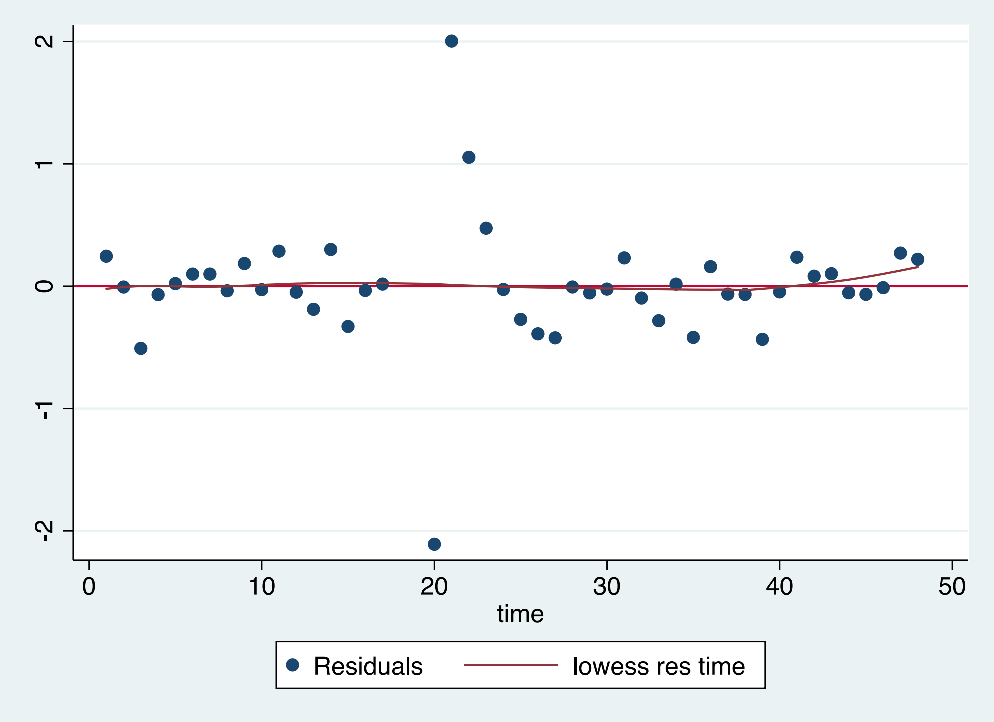

Figure 2b.

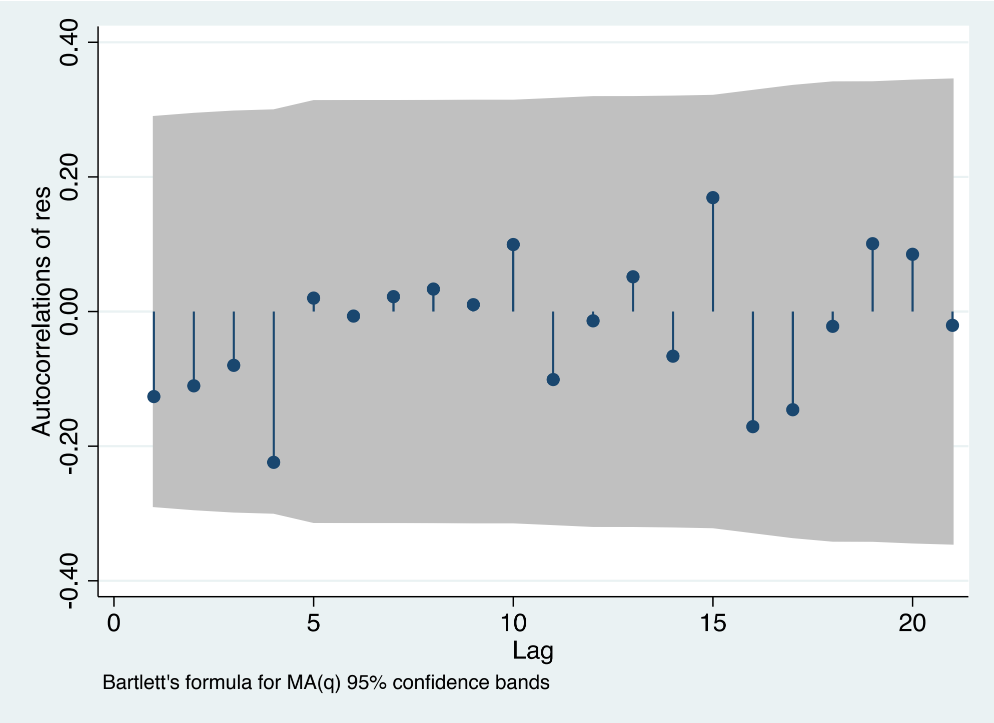

Figure 2c.

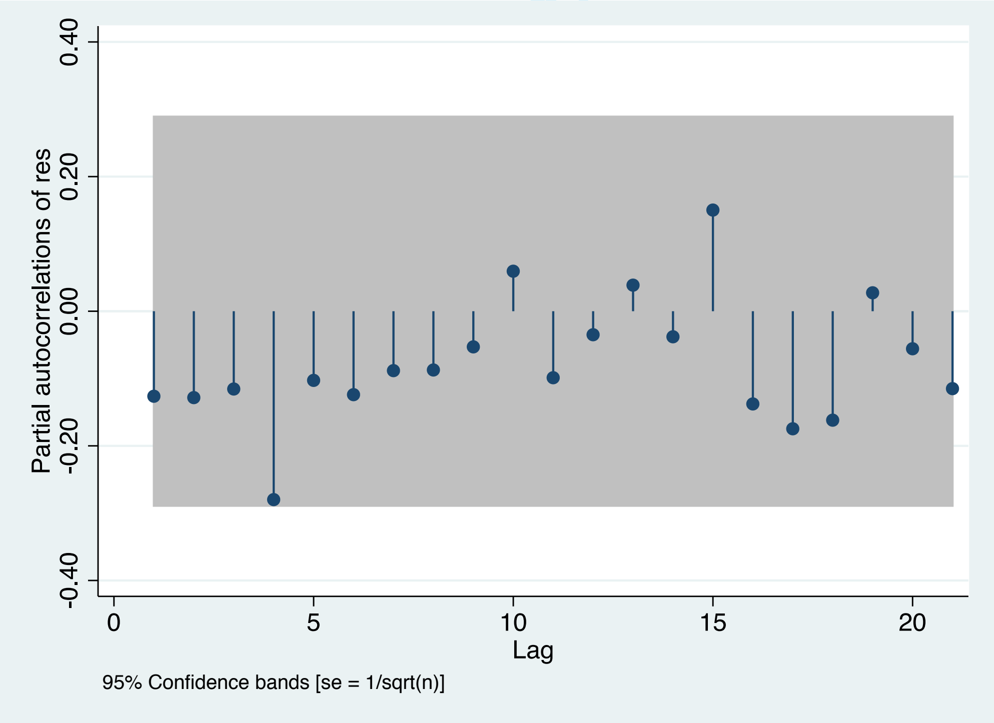

Durbin-Watson d-statistic(4, 46) = 2.243466
